# Star-PAP controlled alternative polyadenylation coupled poly(A) tail length regulates protein expression in hypertrophic heart

**DOI:** 10.1093/nar/gkz875

**Published:** 2019-10-10

**Authors:** A P Sudheesh, Nimmy Mohan, Nimmy Francis, Rakesh S Laishram, Richard A Anderson

**Affiliations:** 1 Cardiovascular and Diabetes Biology Group, Rajiv Gandhi Centre for Biotechnology, Trivandrum-014, India; 2 Manipal Academy of Higher Education, Manipal 576104, India; 3 School of Medicine and Public Health, University of Wisconsin, MD 53726, USA

## Abstract

Alternative polyadenylation (APA)-mediated 3′-untranslated region (UTR) shortening is known to increase protein expression due to the loss of miRNA regulatory sites. Yet, mRNAs with longer 3′-UTR also show enhanced protein expression. Here, we identify a mechanism by which longer transcripts generated by the distal-most APA site leads to increased protein expression compared to the shorter transcripts and the longer transcripts are positioned to regulate heart failure (HF). A Star-PAP target gene, *NQO1* has three poly(A) sites (PA-sites) at the terminal exon on the pre-mRNA. Star-PAP selects the distal-most site that results in the expression of the longest isoform. We show that the *NQO1* distal-specific mRNA isoform accounts for the majority of cellular NQO1 protein. Star-PAP control of the distal-specific isoform is stimulated by oxidative stress and the toxin dioxin. The longest *NQO1* transcript has increased poly(A) tail (PA-tail) length that accounts for the difference in translation potentials of the three *NQO1* isoforms. This mechanism is involved in the regulation of cardiac hypertrophy (CH), an antecedent condition to HF where NQO1 downregulation stems from the loss of the distal-specific transcript. The loss of NQO1 during hypertrophy was rescued by ectopic expression of the distal- but not the proximal- or middle-specific *NQO1* mRNA isoforms in the presence of Star-PAP expression, and reverses molecular events of hypertrophy in cardiomyocytes.

## INTRODUCTION

Almost all eukaryotic mRNAs are polyadenylated at the 3′-end in a coupled process - endonucleolytic cleavage at the PA-site followed by the addition of a PA-tail of up to 250 adenosine residues in the nucleus (1–3). Canonical poly(A) polymerases (PAP) α/γ are involved in the general polyadenylation of nuclear mRNAs (1,4). Identification of a nuclear non-canonical PAP, Star-PAP (Speckle targeted PIPKIα regulated PAP) indicated the existence of selective polyadenylation in the nucleus (5). Although Star-PAP shares structural similarities with non-canonical PAPs, it functions like a canonical PAP but with a distinct mechanism of action (1,5,6). Star-PAP forgoes the use of some of the cleavage factors involved in the canonical polyadenylation and instead associates with a unique set of factors. These factors include phosphatidyl inositol 4 phosphate 5 kinase type I alpha (PIPKIα) that generates the lipid messenger phosphatidyl inositol-4,5-bisphosphate (PI4,5P_2_), RNA binding motif protein 10 (RBM10), casein kinase Iα and ϵ (CKIα/ϵ), and protein kinase Cδ (PKCδ) that regulate Star-PAP function (7–11). Star-PAP selectively targets between 30% and 40% of mRNAs involved in oxidative stress response, tumor formation, cell invasion or apoptosis downstream of diverse cellular signals. The precise fraction of mRNAs targeted by Star-PAP likely depends upon the cell type and signals that are impacting those cells (5,9–12).

The majority of human genes have more than one polyadenylation site at the 3′-UTR that are used alternately to generate transcripts with variable 3′-UTR length (alternative polyadenylation, APA) (13–15). APA of mRNA alters miRNA-mediated control, protein translation or results in new/truncated proteins (16). Widespread shortening of 3′-UTR has been reported in oncogenic activation, CH, cancer progression, stem cell differentiation, HF, and in tissue-specific gene expression (17–24). Shorter mRNAs are associated with increased protein expression attributed to the loss of miRNA regulation (21,23,25). However, several mRNAs with longer APA isoforms show increased protein levels in the cell (26,27). So far, the mechanism of this discrepancy in the 3′-UTR length and resulting protein levels remains unstudied. Further, the exact mechanism of PA-site selection at the 3′-UTR is still not fully understood. A number of trans-acting proteins including the core cleavage and polyadenylation factors, splicing factors, and several RNA binding proteins of diverse functions are implicated in PA-site selection and APA regulation (28–30). Some of the key factors that regulate PA-site usage pattern includes the cleavage stimulatory factor (CstF-64), U1 snRNP, U2 snRNP auxiliary factor 2 (U2AF2), nuclear poly(A) binding protein (PABPN1), Cleavage and polyadenylation factor subunit hFIP1, and the cleavage factor Im (25 and 68 kDa subunits), and the cytoplasmic polyadenylation element binding protein CPEB1 under different cellular conditions (31–43). We have previously shown differences in the genome-wide PA-site usage between the two canonical PAPs α and γ, and the non-canonical Star-PAP (44).

Star-PAP is involved in a genome-wide APA that results in both shortening and lengthening of 3′-UTRs upon its knockdown (44). *NQO1* is one such alternatively polyadenylated Star-PAP target that requires Star-PAP co-regulator PIPKIα for its expression (5,45). *NQO1* encodes for an antioxidant enzyme NADP(H) Quinone Oxidoreductase 1 that catalyses the two-electron reduction of carcinogenic quinone compounds into the reduced form, hydroquinone (46). NQO1 is critical for the cytoprotection of the cell, and is implicated in a number of diseases including heart disease and cancer (47–49). In the heart, *NQO1* expression is down regulated during CH downstream of transcription factor Nrf2 (50,51). *NQO1* transcribes into three mRNA isoforms with different 3′-UTR lengths, the expression of the longest transcript is stimulated by the toxin dioxin (2,3,7,8 tetrachlorodibenzo-p-dioxin, TCDD) (45). TCDD is also shown to induce hypertrophy and hypertension in the heart (52–54). The mechanisms by which various *NQO1* transcripts are generated, or how TCDD stimulates the *NQO1* distal-specific isoform are not defined.

Here, we report a novel function of APA at the *NQO1* 3′-UTR that produces three distinct mRNA isoforms directly linked to HF. We demonstrate that Star-PAP selects the distal-site, controlling the expression of the longest and predominant *NQO1* transcript in cells. The usage of the distal PA-site at the *NQO1* 3′-UTR, as opposed to the proximal or middle sites, results in increased protein expression. We show that the *NQO1* distal-specific transcript is translationally more active compared to the other *NQO1* shorter isoforms, and that the distal-specific isoform accounts for overall cellular NQO1 protein expression. The distal-specific (longer) transcript is also specifically stabilized under oxidative stress or TCDD treatment resulting in the stimulation of NQO1 protein expression. Our study unravels an earlier undefined mechanism of TCDD-mediated NQO1 stimulation that operates through enhancement of the distal-site specific isoform. We show that differential PA-tail addition accounts for the difference in the expressed protein levels from the three *NQO1* mRNA isoforms. This is confirmed by an *in vitro* translation system using *NQO1* mRNA with a short and long PA-tail. Using cellular and animal models of CH, we demonstrate that NQO1 down regulation is associated with the loss of distal PA-site specific transcript as a result of inherent reduction in Star-PAP level during CH. Expression of NQO1 from the distal- but not proximal- or middle-PA-site encoded transcript rescues hypertrophy-induced NQO1 downregulation, and significantly reverses cellular and molecular events of hypertrophy in the cardiomyocyte. This study establishes a novel APA mechanism through Star-PAP-mediated and differential PA-tail length that plays a role in the regulation of CH.

## MATERIALS AND METHODS

### Cell lines, animals and transfections

HEK 293, HeLa and H9c2 cell lines were obtained from American Type Culture Collection and maintained in DMEM with 10% FBS and penicillin/streptomycin (50 U/ml) at 37°C in 5% CO_2_. siRNA oligos were transfected using calcium phosphate method (55) or Oligofectamine (Invitrogen), and plasmid DNAs using Lipofectamine (Invitrogen) as per manufacturer's instructions.

All animal experiments were approved by Institutional Animal Bioethics Committee (IAEC) and strictly followed the guidelines prescribed for experiments using animal subjects. 8- to 12-week-old male Wistar rats were used for the experiments. Animals were procured and housed in RGCB animal house in a temperature and humidity controlled facility.

### Immunoprecipitation and immunoblot analysis

Immunoprecipitations and immunoblottings were carried out as described previously (56). Input = 10% of the total protein used for IP.

### 3′-RACE assay, 3′-end cleavage measurement and PA-tail sequencing

Total RNAs were isolated from HEK 293 cells using RNAeasy mini Kit (Qiagen). 3′-RACE assays were carried out as described earlier (56). RACE products were confirmed by sequencing. For measurement of cleavage efficacy, uncleaved mRNA levels were measured by quantitative real time PCR (qRT-PCR) using a pair of primers across the cleavage site (57). The non-cleaved messages were expressed as fold-change over the total mRNA.

For sequencing of the PA-tail, mRNA was first decapped using RppH enzyme (NEB) followed by circularization using T4 RNA Ligase (New England Biolabs, Ipswitch, MA, USA) as described earlier (58). This circular RNA was then reverse transcribed using inner gene specific primers followed by a nested PCR amplification using outer gene specific primers. The PCR product was then cloned using TOPO TA Cloning system (Invitrogen) and sequenced using M13 reverse primer.

### Quantitative Real-time PCR (qRT-PCR)

qRT-PCR was carried out in a CFX96 multi-colour system (Bio-Rad) using SYBR Green Supermix as described previously (9) with 2 μg of total RNA reverse transcribed using MMLV reverse transcriptase (Invitrogen). Single-product amplification was confirmed by melt-curve analysis, and primer efficiency was near 100% in all experiments. Quantification is expressed in arbitrary units, and the target mRNA abundance was normalized to the expression of GAPDH with the Pfaffl method. All qRT-PCR results were representative of at least three independent experiments (*n* >3).

### Analysis of mRNA stability

HEK 293 cells were transfected with the reporter *FLAG-NQO1* constructs. Cells were then treated with actinomycin D (5 μg/ml in DMSO) for 0, 1, 2, 4, 6, 8, 12, 16, 24 and 48 h post transfection and harvested for RNA isolation (59). qRT-PCR was performed and half-life (*T*_$\frac{1}{2}$_) was measured as described earlier by following the decrease in % mRNA level over time with 0 time point taken as 100% of each reporter expression (60).

### RNA EMSA, *in vitro* cleavage, and polyadenylation assay

Uniformly radiolabeled *NQO1* 3′-UTR RNA was synthesized by *in vitro* transcription using corresponding DNA construct with different PA-sites under T7 promoter, and EMSA experiments were carried as described earlier (57). Active HeLa nuclear extracts were prepared and cleavage assays were reconstituted using template RNAs employed in the EMSA experiment (encompassing key cleavage and polyadenylation elements) as described earlier (6).

For *in vitro* polyadenylation assays, FLAG-Star-PAP was purified from stably transfected HEK 293 cells using anti-FLAG affinity purification system (Sigma). *In vitro* polyadenylation assay was performed using the 45-mer RNA oligonucleotide (UAGGGA)_5_A_15_ (A_45_) template as described previously (5).

### *In vitro* translation

A truncated *FLAG-NQO1* construct was generated with an overhang of T_15_ followed by restriction sites for SpeI and KpnI in the reverse direction and HindIII site in forward direction. PCR product was cloned directionally to the T7 promoter into pTZ19R vector through HindIII and KpnI sites, linearized using SpeI to generate a 3′-overhang that was later removed by Klenow enzyme resulting in a T end on the construct. *In vitro* transcription was then carried out with the construct to generate a capped FLAG-NQO1 mRNA template with short (A) tail (A_16_) for *in vitro* translation. Long (A)-tail template was generated by *in vitro* polyadenylation of the short (A) template using *Escherichia coli* PAPI. *In vitro* translation was carried out using both short and long (A)-tail RNA templates in active HeLa cytoplasmic extract as described earlier (61).

### Reporter assays

Each *FLAG-NQO1* reporter construct has the *NQO1* coding sequence under pCMV promoter, and the full length *NQO1* 3′-UTR region (∼1950 nt long) or control *SV40* 3′-UTR region at the 3′-end as described earlier (57). To obtain regulation by a specific *NQO1* PA-site (proximal, middle, or distal), the poly(A) signals (PA-signals) of the other two remaining PA-sites were mutated (AAUAAA to AAGTAC) on the construct that abolishes activity of respective PA-sites (6). For example, the *FLAG-NQO1* distal PA-site specific construct has a full length *NQO1* 3′-UTR with mutations of PA-signals of middle and proximal sites on the *NQO1* terminal exon. Reporter expression levels were measured by Western blot using anti-FLAG antibody after transfection, and qRT-PCR using a forward primer from *FLAG* and a reverse primer within the *NQO1* coding sequence (57).

### Hypertrophy induction in H9c2 cell line and Wistar rat heart

To induce cellular hypertrophy, H9c2 cells were treated with isoproterenol (100 μM in DMSO) for 48 h (62). Molecular changes after the treatment were analyzed by qRT-PCR and Western blotting. To induce hypertrophy with TCDD, H9c2 cells were treated with 10 nM TCDD for 48 h (54) unless otherwise indicated in the text. Molecular changes after the treatment were analyzed by qRT-PCR and Western blotting. Phase contrast imaging and immunostaining of H9c2 cells were carried out as described previously (10).

For animal experiments, 8- to 12-week-old rats were administered isoproterenol (15 mg/kg animal body weight) intra-peritoneally for seven consecutive days (63), while control animals were given normal saline. Left ventricular hypertrophy was assessed by echocardiography before animals were sacrificed for tissue analysis.

### Polysome analysis

Polysome profiling was carried out as described earlier (64–66). Briefly, HEK 293 cells transfected with Star-PAP RNAi reagents (56) or different *NQO1* expression constructs, and H9c2 cells, induced with isoproterenol or DMSO were treated with cyclohexamide (100 μg/ml) for 10 min before harvesting as described previously (65). Polysome lysates were then prepared in a pre-chilled polysome extraction buffer (20 mM Tris, pH 7.5, 10 mM MgCl_2_, 250 mM NH_4_Cl, 5 mM DTT and 0.5% (v/v) NP40) for 10 min as described previously (64). The clear lysate was layered on the top of a 10–50% sucrose density gradient prepared in the same polysome extraction buffer and subjected to ultracentrifugation (30000 RPM for 16 h at 4°C, Beckman 50.2 Ti rotor) as described earlier (64). Each fraction of the density gradients were collected by upward displacement and the absorbance was monitored at 254 nm. Ribosomal RNA content measured at 254 nm were plotted against fraction numbers to obtain the polysome profile of each sample. RNA in the fractions was extracted with TRIzol reagent and equal volumes of RNA from each fraction were used for reverse transcription to analyse for *NQO1* mRNA level by qRT-PCR and semi-quantitative RT-PCR as reported earlier (67,68). To obtain the relative (% mRNA) polysomic distribution, polysome fractions (9–14 fractions) were combined and mRNA levels were measured by qRT-PCR. *NQO1* mRNA level in the combined polysomic fractions were normalized to *GAPDH* and expressed as relative of total mRNA (percent) in the fractions.

### RNA sequence and miRNA binding site analysis

Complete *NQO1* 3′-UTR sequence was retrieved from NCBI (Gene Id:1728) and individual 3′-UTR regions were selected as reported (45). The individual 3′-UTR sequences were scanned for valid miRNA regions using miRDB (69). Only miRNAs with seed sequence that has exact matching nucleotides on the *NQO1* UTR, and a target score >80 as suggested in the miRDB were selected. Reg RNA-2 (70) was used to identify functional RNA motifs and sites present within the region. We primarily focused on regulatory sequence elements that would likely affect 3′-UTR-mediated mRNA stability and translatability such as AU-rich elements, ribosome binding sites, PIWI-interacting elements, ncRNA hybridization region, 3′-UTR motifs >20 different elements (71), and secondary structural elements. We considered only those sequences that are conserved in length and have at least 50% similarity in sequence composition with earlier reported regulatory elements/sequences.

### Statistics

All data were obtained from at least three independent experiments. For animal experiments, we used data from at least *n* = 5 independent animals. Data are represented as mean ± standard error mean, SEM. The statistical significance of the differences in the mean is calculated using ANOVA. The differences were considered statistically significant at a *P*-value of <0.05.

### Antibodies and primers

List of all RNA oligos, primers and antibodies are shown in Supplementary data.

## RESULTS

### Alternative polyadenylation at the NQO1 3′-UTR and the role of Star-PAP in the PA-site selection

**Figure 1.**
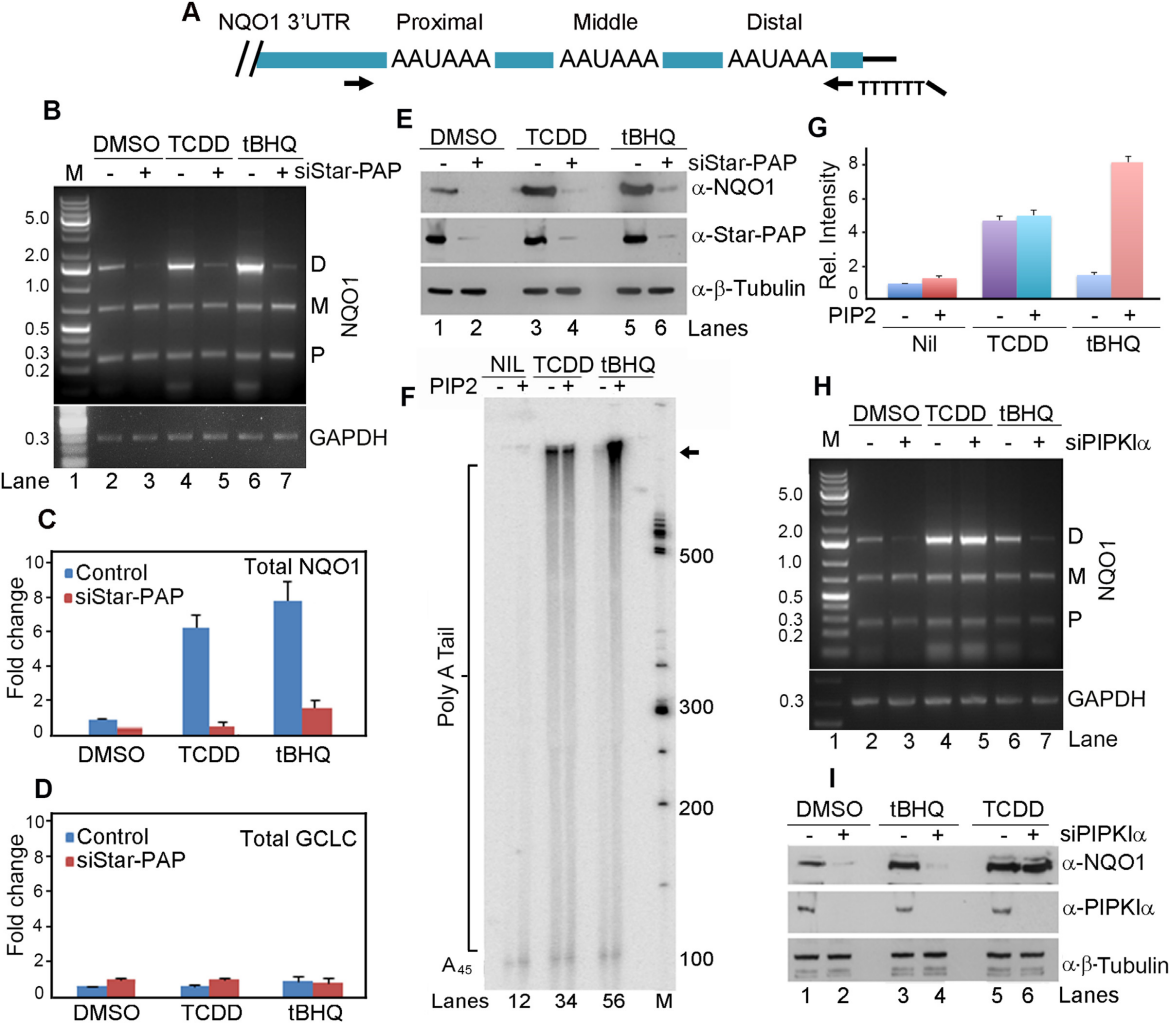
Alternative polyadenylation (APA) at the *NQO1* 3′-UTR generates three mRNA alternate isoforms that is signal regulated. (**A**) Schematic of *NQO1* mRNA 3′-UTR depicting distribution of the three PA-sites (PAS) and the position of the gene specific primer and engineered oligodT primer employed in the 3′-RACE assay. (**B**) 3′-RACE analysis of the PA-site usage within *NQO1* and control *GAPDH* pre-mRNA 3′-UTR in the presence or absence of Star-PAP knockdown after DMSO, tBHQ and TCDD treatment in HEK 293 cells. Products corresponding to the usage of the three PA-sites (proximal, P; middle, M; distal, D) on the *NQO1* 3′-UTR are indicated. Each gel is representative of *n* = 3 independent experiments. (**C, D**) qRT-PCR analysis of *NQO1* and a Star-PAP non-target *GCLC* under conditions as in B (*P*-values - DMSO 0.01, tBHQ 0.045, and TCDD 0.03 for *NQO1*; *P*-values <0.02 for *GCLC* under all three conditions). Error bar represents standard error mean (SEM), (*n* = 3). (**E**) Western blot analysis of NQO1, Star-PAP and control β-tubulin from HEK 293 cells under the same condition as in B. Each blot is representative of *n* = 3 independent experiments. (**F**) *In vitro* polyadenylation assay carried out with the FLAG-Star-PAP complex isolated from stable expressing FLAG-Star-PAP in HEK 293 cells after treatment with DMSO, tBHQ and TCDD using a 45-mer RNA oligonucleotide template having a repeat sequence UAGGGA followed by a stretch of 15 adenosines, (UAGGGA)_5_A_15_ (A_45_) (5) in the presence or absence of PI4,5P_2_ as indicated. Each autoradiogram is representative of *n* = 3 independent experiments. (**G**) Densitometric quantification of intensities of bands in phosphor images of PAP assays in F in arbitrary units expressed as relative intensity with respect to the intensity of FLAG-Star-PAP alone (quantified regions on the gel is indicated by an arrow). Data are mean ± SEM of *n* = 3 independent experiments. (**H, I**) 3′-RACE and Western blot analysis as in B and E respectively but in the presence and absence of PIPKIα knockdown as indicated. Each gel is representative of *n* = 3 independent experiments.

Recently, we reported that Star-PAP regulates genome-wide APA where it preferentially controls the distal PA-sites on the target 3′-UTRs (44). Using 3′-RACE assay, we confirmed the occurrence of the three functional PA-sites on the *NQO1* pre-mRNA 3′-UTR that produces three *NQO1* transcripts (Figure 1A-B). We refer to the three PA-sites as the distal (D), middle (M) and proximal (P) based on their positional proximity to the 5′-end (Figure 1A). Sequence of the three *NQO1* distinct PA-site regions are shown in Supplementary Figure S1. Star-PAP knockdown resulted in a specific loss of the longest distal-specific isoform with no effect on the two shorter proximal- or middle-encoded isoforms or control *GAPDH* 3′-UTR (Figure 1B, lanes 2 and 3) in HEK 293 cells. We further tested Star-PAP mediated APA on additional targets *PTBP2, ANXA7, FOG2* and *PAK1* 3′-UTR (Supplementary Figure S2A-D). We consistently observed a loss of the distal PA-site specific isoform among the two isoforms of *PTBP2* and diminished expression of both proximal and distal PA-site specific isoforms of *ANAX7* upon Star-PAP knockdown (Supplementary Figure S2A-B). On the other hand, we observed a shift in the distal to proximal PA-site usage on *FOG2* upon Star-PAP knockdown (Supplementary Figure S2C). In the case of *PAK1*, we detected four mRNA isoforms of which Star-PAP knockdown resulted in the reduction of the last two distal-site specific isoforms with no effect on the two remaining proximal sites (Supplementary Figure S2D). Quantitative real-time PCR (qRT-PCR) indicated less than 40% reduction of the total endogenous *NQO1* mRNA level (Figure 1C, DMSO) on Star-PAP knockdown with no effect on the non-target *GCLC* (Figure 1D). However, the same knockdown resulted in a near-complete loss of the NQO1 protein (Figure 1E, lanes 1 and 2) indicating that the distal-specific isoform is the predominant transcript for cellular NQO1 protein expression, and that Star-PAP controls overall NQO1 protein expression. Significantly, this finding demonstrates a direct effect of 3′-UTR length on the resultant protein expression of the APA generated isoforms as reported earlier (21–23,26,72).

### NQO1 distal site usage is signal regulated

NQO1 expression is stimulated by the oxidative stress agonist tBHQ, and the toxin dioxin (TCDD) that also induces CH and hypertension in the heart (45,53,73). To investigate how these two signals impact *NQO1* APA, HEK 293 cells were stimulated with tBHQ (100 μM) for 4 h (5) or TCDD (100 nM) for 24 h (45), and APA was analyzed by 3′-RACE assay. Both tBHQ and TCDD specifically stimulated generation of the distal-specific transcript, an effect that was dependent on Star-PAP (Figure 1B, lanes 4–7). Similarly, NQO1 total mRNA and protein expressions were also increased upon tBHQ and TCDD treatment in a Star-PAP-dependent manner (Figure 1C, E, lanes 3–6). To confirm this finding, we purified FLAG-Star-PAP from stable FLAG-Star-PAP expressing HEK 293 cells after treatment with TCDD or tBHQ, and reconstituted *in vitro* polyadenylation assays. We observed that tBHQ treatment stimulated Star-PAP activity in the presence of PI4,5P_2_ as reported earlier (5) (Figure 1F). TCDD treatment also stimulated Star-PAP polyadenylation activity but did not prime Star-PAP for further stimulation by PI4,5P_2_ (Figure 1F-G). This indicated a distinct pathway for TCDD stimulation of Star-PAP toward *NQO1* compared to oxidative stress stimulation of Star-PAP activity. Consistently, the knockdown of PIPKIα (that synthesises nuclear PI4,5P_2_) resulted in a reduced expression of the basal and tBHQ-stimulated expression of *NQO1* distal-specific isoform, but did not affect the TCDD-stimulated expression (Figure 1H). Knockdown of PIPKIα also resulted in the loss of unstimulated and tBHQ-stimulated but not the TCDD-stimulated NQO1 expression (Figure 1I). Knockdown of another Star-PAP co-regulator casein kinase Iα (74), a PI4,5P_2_ effector that couples with PIPKIα for Star-PAP regulation exhibited a similar loss of the distal-specific *NQO1* isoform, and a concomitant loss of the unstimulated- and tBHQ-stimulated NQO1 protein levels (Supplementary Figure S2E-F) similar to PIPKIα knockdown. Thus, TCDD or tBHQ mediated induction of distal-specific *NQO1* transcript stimulates overall NQO1 protein expression through regulation of Star-PAP but by distinct signaling mechanisms. These results illustrate a signal-regulated APA site usage at the *NQO1* 3′-UTR, and also define a hitherto unknown mechanism of TCDD-mediated NQO1 stimulation through the distal PA-site selection by Star-PAP.

### *NQO1* distal-specific isoform is the primary transcript for cellular NQO1 protein expression

**Figure 2.**
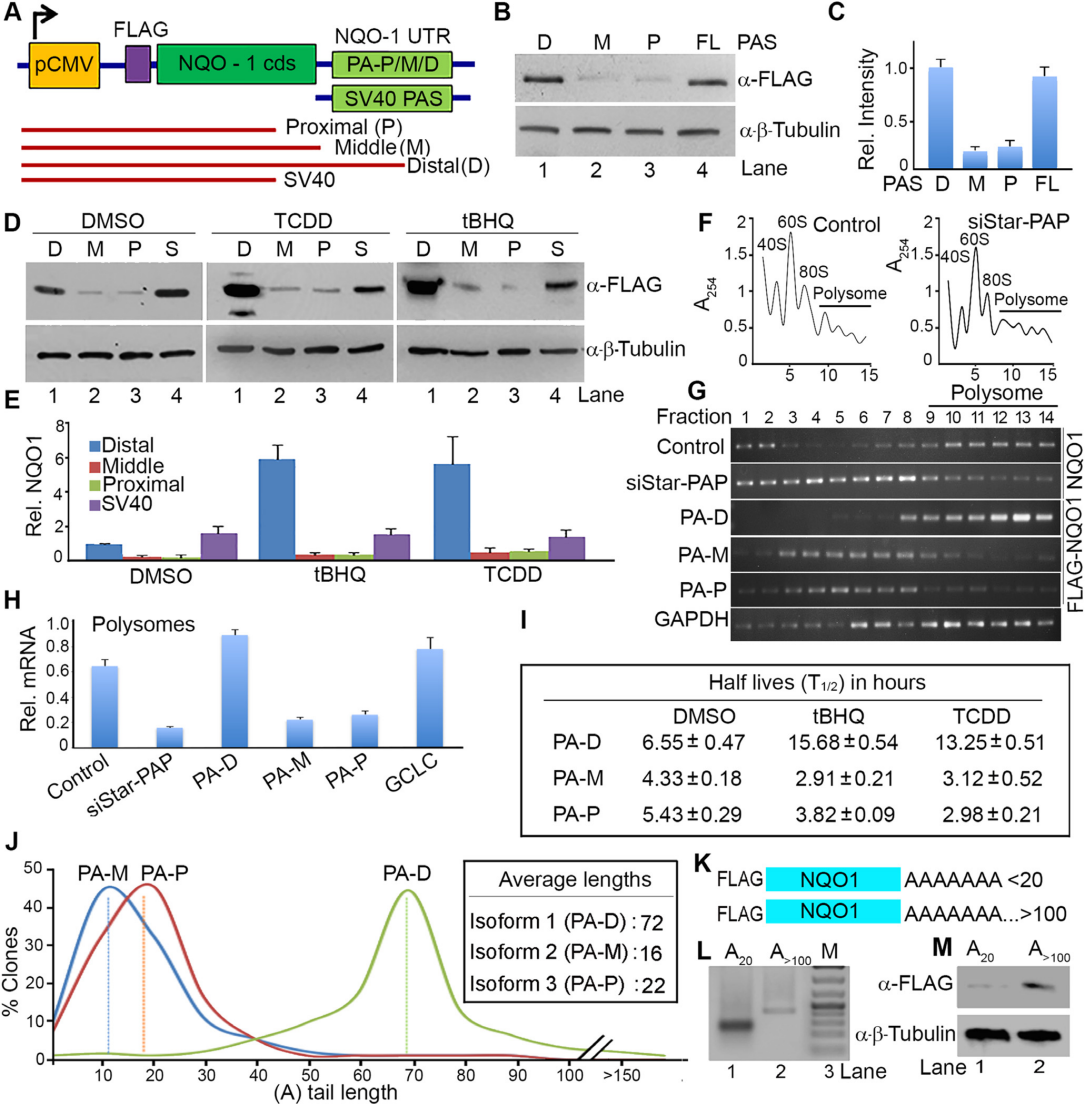
*NQO1* APA generates mRNA isoforms with different translation efficiencies that is linked with differential PA-tail length addition. (**A**) Schematic of a reporter mini gene construct of FLAG-NQO1 expressed from the CMV promoter and driven by each *NQO1* PA-site (P-proximal, M-middle, and D-distal) or control *SV40* 3′-UTR. Sequences of respective 3′-UTRs are shown in Supplementary Figure S1. Red lines represent *FLAG-NQO1* mRNA isoforms generated from the proximal, middle, or distal PA-site specific reporter constructs. (**B, C**) Western blot analysis and quantification of FLAG-NQO1 from HEK 293 cells after transfection of the three *FLAG-NQO1* mini-gene reporter constructs. Quantification of band intensity was performed by using Image J software and is shown in C. Intensities (in arbitrary units) for each band was normalized with β-tubulin and expressed as fold difference in relative intensity with respect to the control DMSO treated sample. Error bar represents SEM of *n* = 3 independent experiments. (**D, E**) Western blot analysis and quantification of FLAG NQO1 after transfection of different *FLAG-NQO1* reporter mini-gene constructs driven by the three PA-sites (distal-D, middle-M, proximal-P) in the presence and absence of tBHQ (100 μM for 4 h) or TCDD (100 nM for 24 h) treatment as indicated. Quantification of the blot is shown in E. Intensities (in arbitrary units) for each band was normalized with β-tubulin and expressed as fold-difference in relative intensity with respect to the distal-site DMSO treated sample. Error bar represents SEM of *n* = 3 independent experiments. (**F**) Representative polysome profile of HEK 293 cells in the presence and absence of Star-PAP knockdown after fractionation with 10–50% sucrose density gradient of 15 fractions. Ribosomal RNA content measured at 254 nm was plotted against fraction numbers. The top of the gradient is on the left, peaks representing monosomal fractions, and polysomal fractions are indicated. (**G**) RNA isolated from each fraction from the density gradient was analyzed for polysomal association of *FLAG-NQO1* (transfected reporter *NQO1*), endogenous *NQO1*, and *GAPDH* mRNA (from control sample) by semi-quantitative RT-PCR. (**H**) qRT-PCR analysis endogenous *NQO1* mRNA or *FLAG-NQO1* mRNA in the combined polysomic fractions of the density gradient (9–14 fractions). *NQO1* or control *GCLC* mRNA levels normalized to *GAPDH* are expressed relative (percent) of total mRNA. Error bar represents SEM of *n* = 3 independent experiments (*P*-values: 0.003, 0.01, 0.008, 0.03, 0.02, and 0.006 for control, siStar-PAP, PA-D, PA-M, PA-P, and *GCLC* respectively). (**I**) Half-life (T_$\frac{1}{2}$_) measurement of each *NQO1* isoform encoded by proximal (P), middle (M) or distal (D) PA-sites after transcription inhibition with actinomycin D under conditions as indicated. T_$\frac{1}{2}$_ is expressed in hours. Data are mean ± SEM of *n* = 3 independent experiments. (**J**) PA-tail length distribution of *NQO1* mRNA isoforms encoded by distal, middle or proximal PA-sites (sequenced from >200 clone each), and average PA-tail length is indicated in the inset. (**K**) Schematics of the RNA template for *in vitro* translation generated from distal PA-site with short A (A_16_) and long A (>A_100_, after polyadenylation of the A_16_*NQO1* template). (**L**) Agarose gel analysis of both short and long A *NQO1* RNA templates as in K. (**M**) *In vitro* translation using HeLa cytoplasmic lysates with *in vitro* transcribed *NQO1* RNA template generated from the distal PA-site with short A (A_16_) and long A (>A_100_, after polyadenylation of the A_16_*NQO1* template).

To further explore the regulation of *NQO1* APA, we generated reporter mini-gene constructs where FLAG-tagged *NQO1* coding sequence (cds) transcribed from the CMV promoter was controlled by different PA-sites on the 3′-UTR of *NQO1* (proximal, middle and distal), or *SV40* control (57) as detailed in materials and methods (Figure 2A). Reporter expression was measured by Western blot using anti-FLAG antibody or by qRT-PCR as reported earlier (57). We validated *NQO1* APA, stimulation by tBHQ and TCDD of the distal-specific isoform, and the Star-PAP dependent expression of the distal-site specific isoform using the *FLAG-NQO1* construct (Supplementary Figure S2G-H). There was a loss of NQO1 expression from the distal- but not proximal- or middle-specific constructs on Star-PAP knockdown, and subsequent stimulation by tBHQ or TCDD treatment (Supplementary Figure S2H-I). But expression of FLAG-NQO1 from SV40 PA-site was robust and was not affected by either Star-PAP knockdown or tBHQ/TCDD treatment (Supplementary Figure S2H-I). Next, the *FLAG-NQO1* reporter constructs were transfected into HEK 293 cells and the expression of NQO1 protein from each of the three isoforms was quantified. There was greater expression of FLAG-NQO1 protein (>6-fold) from the distal-specific isoform compared to the proximal- or middle-specific isoforms (Figure 2B-C). This finding is consistent with the loss of NQO1 expression on Star-PAP knockdown and demonstrates that Star-PAP has specificity for the distal PA-site. The NQO1 expression from the distal PA-site was also robustly stimulated by TCDD and tBHQ treatment (∼6-fold difference between the distal- versus the middle- or proximal-encoded transcripts) (Figure 2D-E). These results indicate that the *NQO1* distal-specific isoform is the primary *NQO1* transcript responsible for the cellular NQO1 protein expression.

### *NQO1* distal PA-site specific isoform is translationally more active than the two shorter isoforms

The translation potentials of the three *NQO1* APA isoforms were quantified by polysome profile analysis of the actively translating and non-translating mRNA fractions in HEK 293 cells (67,68). For this, HEK 293 cells were transfected with siRNA specific to Star-PAP or the *NQO1* reporter constructs, and fractionated by sucrose density ultracentrifugation as described earlier (64,65). There was no difference in the fractional distribution of polysomes on Star-PAP knockdown (Figure 2F) or on transfection. While non-polysomes (free RNA, 40S, 60S and 80S ribosomes) were detected in fractions 1 to 8, polysomes were observed largely from 9 to 14 fractions of the density gradient (Figure 2F). We then analyzed *NQO1* mRNA distribution in each fraction from the density gradient using qRT-PCR and semi-quantitative RT-PCR as described earlier (67,68). Strikingly, there was a significant difference in the distribution of the three *NQO1* isoforms in the density gradient profiles (Figure 2G). The distal-specific *NQO1* isoform was predominantly detected in the polysomic fractions, whereas the middle and proximal driven *NQO1* transcripts were distributed largely in the non-polysomal fractions (Figure 2G). Endogenous *NQO1* mRNA, too, was broadly distributed throughout the polysome portion of the gradient similar to control GAPDH (Figure 2G). Further, there was a reduction of *NQO1* mRNA level in the polysomal portion of the gradient upon Star-PAP knockdown consistent with the reduced NQO1 protein level on the knockdown (Figure 2G). More than 65% of *NQO1* mRNA was observed in the polysomic fraction of the gradient that was reduced to ∼17% on the Star-PAP knockdown (Figure 2H). Greater than 90% of the distal-specific transcript was found in the polysomal portion of the gradient (9–14 fractions), compared to ∼20% of the middle- or proximal-specific transcripts in these fractions (Figure 2H). These results demonstrate that the distal-specific isoform is translationally more active compared to the two shorter isoforms.

To further understand the difference in the translatability of the three *NQO1* mRNA isoforms, we measured the stability of the transcripts after actinomycin D treatment. There was no marked difference in half-lives (*T*_$\frac{1}{2}$_) among the three isoforms (Figure 2I) without stimulation. While *T*_$\frac{1}{2}$_ of the longest *NQO1* distal-specific isoform was ∼6.5 h, the shorter middle- and proximal-specific isoforms had *T*_$\frac{1}{2}$_ of ∼4.3 and ∼5.4 h respectively (Figure 2I). Stimulation with tBHQ or TCDD specifically enhanced stability of the longest distal-specific isoform by >2-fold with a modest reduction in the stability of the middle- and proximal-specific isoforms (Figure 2I). This is consistent with our observed increased NQO1 protein expression upon tBHQ or TCDD treatment. Together these results indicate that the distal-specific *NQO1* mRNA isoform is translationally more active than the middle- or proximal-specific isoforms, and accounts for overall basal NQO1 protein expression. The increased stability of the distal isoform accounts for the signal-induced NQO1 expression upon TCDD or tBHQ treatment.

### PA-tail length controls NQO1 protein of the distal APA isoform

To investigate the mechanism for enhanced expression of the longest *NQO1* mRNA isoform, we analyzed miRNA binding sites and putative unique RNA regulatory elements (AU-rich elements, PIWI-interacting elements, ncRNA hybridization region, 3′-UTR motifs such as Mushashi elements, and secondary structural elements) across the three *NQO1* PA-sites at the 3′-UTR. No specific regulatory sequence elements (AU-rich element, ribosome binding site, secondary structural elements) were found that could affect the stability and translation efficiencies of these transcripts (Supplementary Figure S3A). There were few potential miRNA target sites (1 upstream of the proximal-, 6 upstream of the middle- and 4 upstream of the distal-site) (Supplementary Figure S3A). This did not explain how the longer *NQO1* transcript has higher protein expression than the two shorter isoforms in the cell. Therefore, we sequenced PA-tails of endogenous transcripts of each *NQO1* isoform by cloning into TA-cloning vectors after c-RACE analysis (Figure 2J) (58). Intriguingly, we discovered the median PA-tail length of the distal isoform was ∼72 As compared to ∼22 and ∼16 As of the shorter isoforms (Figure 2J). The majority of clones had PA-tail length ranging from ∼50 to ∼90 for the distal-specific *NQO1* transcript while the other two isoforms ranged upto ∼30 As (Figure 2J). We further confirmed the PA-tail length of each isoform by sequencing after transient expression of respective reporter constructs and observed similar length differences among the three isoforms (data not shown) suggesting that the higher NQO1 expression is regulated by the PA-tail length.

PA-tails are critical for the translation of an mRNA and the prevailing view is that longer A-tails enhances translation efficiency (75,76). Recently, with high throughput PA-tail sequencing, the concept of coupling of PA-tail length with translation became a matter of debate (77–79). Nevertheless, requirement of longer A-tail for efficient translation was demonstrated in earlier studies using *in vitro* translation systems, and/or cellular reporter assays (61,80). To confirm our finding, we transcribed *FLAG-NQO1* distal-specific RNA *in vitro* with two different (A)-lengths (short with 16 As and long with >100 As) (Figure 2K-L). We then carried out *in vitro* translation using active HeLa cytoplasmic extracts (61) and detected proteins synthesized using anti-FLAG antibody. We observed higher NQO1 protein from the transcript with longer (A)-tail (>3-fold) compared to the shorter (A) tail (Figure 2M) while we detected similar amounts of residual mRNA templates in the two reactions (Supplementary Figure S3B). Further, we treated HEK 293 cells after transfection of the distal PA-site specific *FLAG-NQO1* reporter construct with the PA-chain terminating agent cordycepin that reduces PA-tail length in the cell (81,82). We observed decreased FLAG-NQO1 protein level with increasing time of the cordycepin treatment consitent with reduced A-tail length (Supplementary Figure S3C) as reported earlier (82,83). These results demonstrate that longer PA-tail lengths of *NQO1* distal-specific mRNA isoform are associated with higher NQO1 protein levels. This re-establishes that PA-tail length is coupled with translation of an mRNA for the *NQO1* APA-generated transcripts.

### Star-PAP specificity for the distal PA-site on *NQO1* controls efficient processing

**Figure 3.**
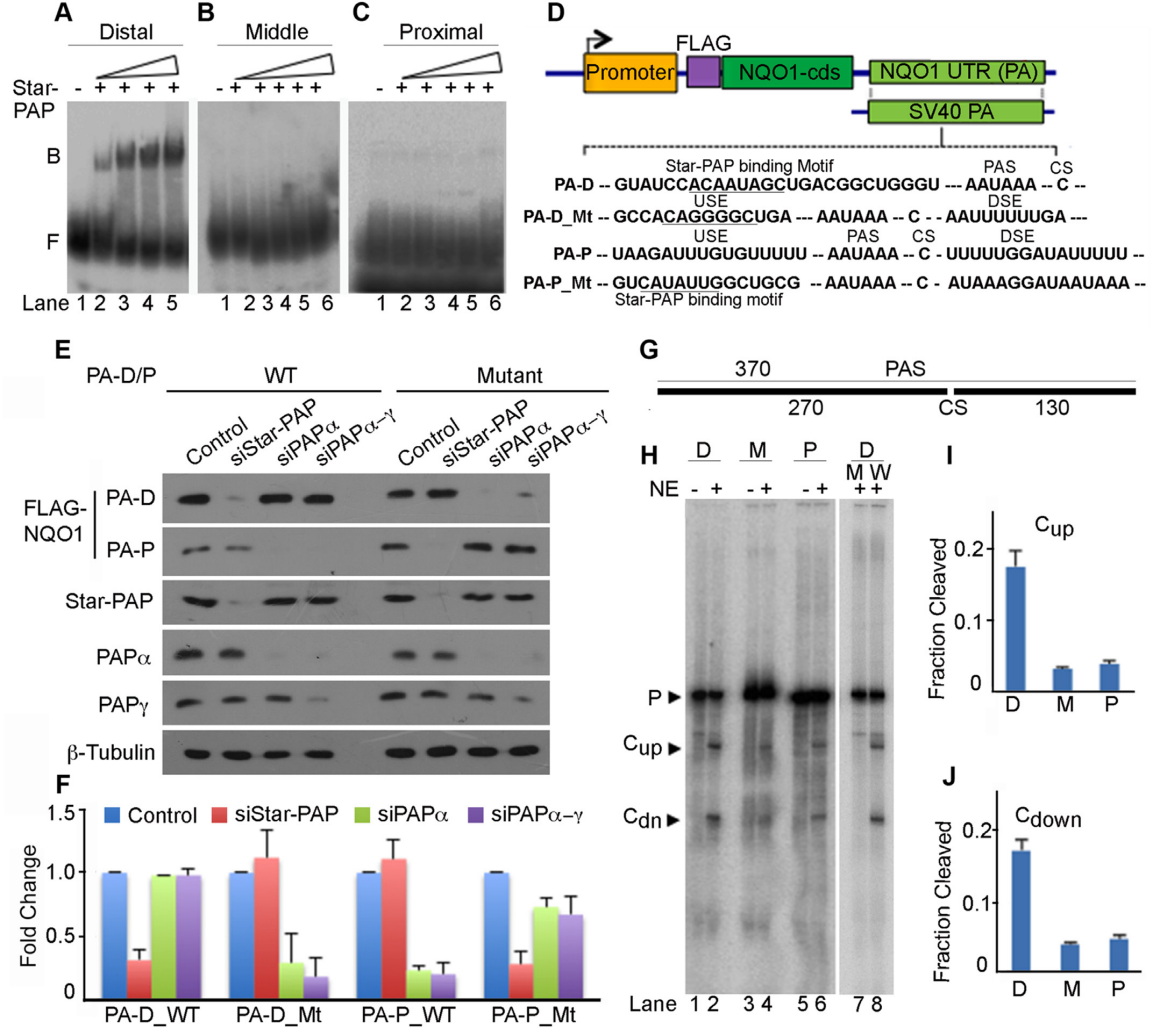
Star-PAP specifically selects the distal PA-site at the *NQO1* 3′-UTR and is processed more efficiently. (**A–C**) RNA EMSA using 3′-UTR fragments with each *NQO1* PA-site and recombinant His-Star-PAP. Binary complex, B and Free probe, F are indicated. (**D**) Schematic of *NQO1* reporter constructs and mutations introduced on the distal (PA-D) and proximal (PA-P) sites as indicated. Sequence of PA sites and mutations are indicated in the Supplementary Figure S1. (**E**) Western blot analysis of FLAG-NQO1 expressed from reporter constructs driven by distal (PA-D) and proximal (PA-P) PA-sites where mutations were introduced at the downstream and upstream sequences relative to respective PA-sites as indicated. Each blot is representative of *n* = 3 independent experiments. (**F**) qRT-PCR analysis of reporter *FLAG-NQO1* under the condition as in E. Error bar represents SEM (*n* = 3) independent experiments (*P*-value <0.04 for PA-D_WT, <0.02 for PA-D_Mt and PA-P_Mt, <0.01 for PA-P_WT). (**G**) Schematics of 3′-UTR RNA template for *in vitro* cleavage template. (**H–J**) *In vitro* cleavage assay using respective 3′-UTR fragments with each PA-site (D-distal, M-middle, P-proximal) and PA-signal mutation (W-wild type, M-AAUAAA to AAGTAC mutation,) of the distal-site 3′-UTR as indicated. Schematic of the template is depicted in G, autoradiogram of the experiment in H, and quantifications in I–J. Quantifications of both pre-mRNA (P) and cleaved (upstream, C_up_ and downstream, C_Dn_) fragments were carried out using imageJ software and relative intensities (in arbitrary units) for cleaved fragments were expressed as fraction cleavage relative to total intensity of both uncleaved and cleaved fragments. Error bar represents SEM, *n* = 3 independent experiments. Upstream fragment (C_up_) and downstream fragment (C_Dn_) after the cleavage of the template are indicated.

To define how Star-PAP controls the distal PA-site on the *NQO1* 3′-UTR, the sequence around the three PA-sites was analyzed. Star-PAP recognises 3′-UTRs with a GC-rich sequence having an -AUA- motif ∼40–60 nucleotide upstream of the PA-site, and a U-deplete sequence (suboptimal DSE) downstream of the PA-site (6,9,57). Analysis of the sequence composition around each PA-site indicated higher GC-content (within 100 nucleotides) upstream of the distal-site along with an -AUA- motif (∼40 nucleotides upstream) compared to the other two PA-sites on the *NQO1* 3′-UTR (Supplementary Figures S1, S4A). A low U region at the DSE of the *NQO1* distal site was observed (Supplementary Figure S4B) similar to other known Star-PAP targets *HO-1* and *BIK* mRNA 3′-UTRs (6,9). To confirm this usage, quantitative RIP analysis was carried out to demonstrate the selective association of Star-PAP with the *NQO1* distal-site (Supplementary Figure S4C). Using *in vitro* transcribed mRNA templates for each *NQO1* PA-site (Supplementary Figure S1), further illustrated that Star-PAP specifically binds the distal-site and not the middle- or proximal-site (Figure 3A-C).

To investigate Star-PAP specific selection of the distal PA-site, we introduced a U-rich DSE (UUUUUU) to make the site proficient for CstF-64 recognition, and a Star-PAP binding mutation in the distal-specific reporter construct (57) to make it a non-Star-PAP target PA-site (Figure 3D). Knockdown of Star-PAP resulted in the loss of wild-type reporter expression in HEK 293 cells with no effect of either PAPα or PAPα/γ knockdowns (57) (Figure 3E-F). But, expression from the mutant construct was not affected by Star-PAP knockdown but was lost when the canonical PAPs were knocked down. We also used the proximal-specific construct and introduced mutations in the DSE (to make it suboptimal for CstF-64 binding) and a Star-PAP binding motif upstream of the PA-signal (Figure 3D) to convert into a Star-PAP dependent PA-site. Interestingly, while FLAG-NQO1 expression for the wild-type reporter construct was independent of Star-PAP, the mutant construct showed significant reduction upon Star-PAP knockdown (Figure 3E-F). Moreover, changing to a Star-PAP dependent PA-site also resulted in a higher protein expression from the proximal-site encoded transcript compared to the wild type (Figure 3E-F). These results show that Star-PAP specifically regulates the *NQO1* distal-site and that by changing the 3′-UTR sequence, the regulatory PAP could be altered (57). This supports our earlier model of 3′-UTR-sequence driven PAP selection (1) and defines a mechanism of *NQO1* APA through Star-PAP mediated selection of the distal-site.

Star-PAP is required for both cleavage of the pre-mRNA and polyadenylation (6). In this context, *in vitro* cleavage assay using active HeLa nuclear extract (6) and RNA templates for each PA-site (Figure 3G, Supplementary Figure S1) revealed a higher cleavage efficiency of the distal-site containing 3′-UTR RNA compared to the templates containing the middle- or proximal-sites (Figure 3H-J). This demonstrated a >10-fold difference in the cleavage efficiency between the distal-site and the middle- or proximal-site (Figure 3H-J) and there was loss of the cleavage when the control PA-signal was mutated from AAUAAA to AAGTAC in the distal-site as described before for other targets (6) (Figure 3H). Loss of Star-PAP specifically affected the cleavage from the distal-site (Supplementary Figure S4D-E) consistent with the *in vivo* experiments that indicate the dominance of the distal-site on the *NQO1* 3′-UTR. These combined results show that *NQO1* expression is controlled largely through the distal PA-site via Star-PAP that results in a lengthy but translationally efficient transcript with longer PA-tail. As NQO1 is an enzyme that is fundamental to many physiological conditions including HF (50,51), we set out to determine if Star-PAP control of *NQO1* APA is regulated during CH which is a component of HF.

### *NQO1* APA is involved in the regulation of cardiac hypertrophy

**Figure 4.**
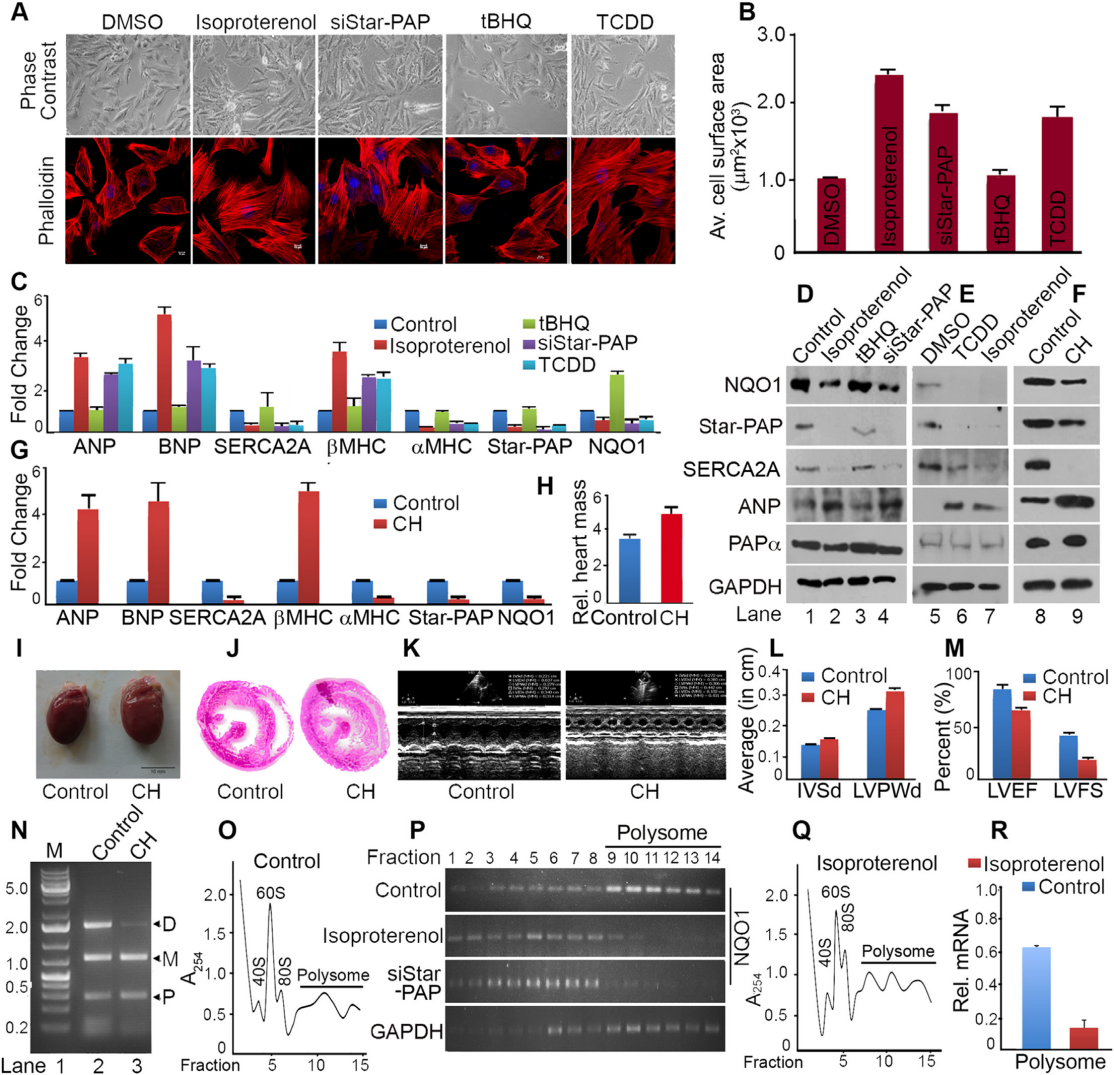
*NQO1* APA is involved in the regulation of cardiac hypertrophy. (**A**) Phase contrast (PC) and IF imaging of phalloidin stained H9c2 cells after treatment with isoproterenol (100 μM for 48 h), TCDD (10 nM for 48 h), control tBHQ (100 μM for 4 h) or solvent DMSO, and siRNA knockdown of Star-PAP as indicated. (**B**) Quantification of cell surface area of the phalloidin stained H9c2 cells. Average cell surface area was measured for >50 cells per experiment for n>3 independent experiments (*P* value = 0.02, 0.04, 0.01, 0.008, 0.03 respectively for DMSO, isoproterenol, tBHQ, siStar-PAP and TCDD treated samples. Error bar represents SEM. (**C**) qRT-PCR analysis of molecular markers of hypertrophy, *NQO1* and *Star-PAP* after treatment with isoproterenol, TCDD, control tBHQ or solvent DMSO, and siStar-PAP as indicated. mRNA levels are expressed relative to DMSO treated samples (*P*-values *ANP* <0.04, *BNP* <0.025, *SERCA2A* <0.03, *b-MHC* <0.04, *a-MHC* <0.005, *Star-PAP* <0.01, and *NQO1* <0.008). Error bar represents SEM (*n* = 3 independent experiments). (**D, E**) Western blot analysis of various hypertrophic markers, Star-PAP, and NQO1 after induced hypertrophy in H9c2 cell line by isoproterenol treatment or TCDD treatment as in C. Each blot is representative of *n* = 3 independent experiments. (**F**) Western blot analysis of Star-PAP, NQO1 and hypertrophic markers from control rat heart and isoproterenol induced hypertrophic rat heart (*n* = 5 independent animals). (**G**) qRT-PCR analysis of corresponding mRNA expressions as in F from control and hypertrophic animal hearts (*n* = 5 independent animals in each group). mRNA levels in hypertrophic heart are expressed relative to control animals (*P*-values: *ANP* - 0.005, *BNP -* 0.04, *SERCA2A* - 0.03, *β-MHC* - 0.01, *α-MHC* - 0.009, *Star-PAP* - 0.04, and *NQO1* - 0.02). Error bar represents SEM. (**H**) Comparison of relative heart mass (heart mass in mg over body mass in gm) ratio of control and hypertrophic heart (*n* = 5 in each group). Error bar represents SEM. (**I, J**) Comparison of the heart size and the cross section of control versus hypertrophic heart. (**K**) Echocardiography analysis of control and hypertrophic animal heart. (**L**) Measurement of interventricular septum diastole (IVSd) and left ventricular posterior wall thickness of control and hypertrophic animals (*n* = 5 per group; *P* values <0.01 for control and <0.005 for hypertrophic heart). Error bar represents SEM. (**M**) Measurement of left ventricular ejection fraction (LVEF %) and left ventricular fraction shortening (LVFS %) of control and hypertrophic animals (*n* = 5 per group, *P* value <0.02 for control and <0.04 for hypertrophic heart). Error bar represents SEM. (**N**) 3′-RACE assay of *NQO1* APA from control and hypertrophy samples from isoproterenol induced hypertrophy in H9c2 cells. (**O–R**) Representative polysome profiles of H9c2 cells with or without isoproterenol treatment after fractionation with 10–50% sucrose density gradient as in Figure 2F. RNA isolated from each fraction was analyzed for the polysomal distribution of *NQO1* or control *GAPDH* mRNA by semi quantitative RT-PCR, and qRT-PCR to measure relative distribution of *NQO1* mRNA (% mRNA relative of total NQO1 mRNA) in the combined polysome fractions.

There is a widespread change in the APA-pattern that affects 3′-UTR length and the resultant protein expression of several regulators during hypertrophy and cardiac remodelling (22,24). NQO1 is a key cytoprotective enzyme in the heart that is reduced during CH downstream of transcription factor Nrf2 (50,51). In addition, the *NQO1* distal specific isoform is induced by TCDD, a chemical that is a risk factor for CH and HF (53). We used both cellular (H9c2 rat cardiomyoblast cells) (62) and an *in vivo* Wistar rat heart model of CH (63) to study the role of *NQO1* APA in CH. Hypertrophy was induced in H9c2 cells by isoproterenol treatment for 48 h (62) and confirmed by checking cellular changes such as increase in cell size or actin filament re-modelling (Figure 4A-B), and molecular changes during hypertrophy (increased expression of foetal genes *ANP, BNP*, and *β-MHC*; and reduced expression of adult gene *α-MHC* and *SERCA2A*) (Figure 4C-D). We observed a loss of Star-PAP as reported earlier (10) and a similar down regulation of NQO1 in the isoproterenol treated cardiomyocytes with a parallel decrease in *SERCA2A* or *α-MHC*, and stimulation of *ANP, BNP* or *β-MHC* (Figure 4C-D). Since TCDD induces hypertrophy of cardiomyocyte (54), TCDD-induced hypertrophy was assessed in H9c2 cells by treatment with 10 nM TCDD for 48 h (54). Induced-hypertrophy was evidenced from increased *ANP, BNP, β-MHC* or decreased *SERCA2A, α-MHC* expression, and increased cell size (∼1.95-fold) compared to DMSO treated cells (Figure 4A-B, E). There was a decrease in mRNA and protein expression of NQO1 and Star-PAP under TCDD-induced hypertrophy similar to that in isoproterenol-induced hypertrophy (Figure 4A-B,E). We also observed similar hypertrophic response on treatment of H9c2 cells for longer time period of 72 h with the same amount of TCDD (Supplementary Figure S5A-C).

TCDD is a ligand activator of Aryl hydrocarbon receptor, Ahr that induces dioxin/xenobiotic response element (AHRE/DRE/XRE) containing genes including those drug metabolizing and detoxification enzymes (84,85). Activation of the drug metabolizing enzymes causes production of reactive oxygen species (ROS) that leads to oxidative stress and subsequent cardiac toxicity and hypertrophy in the heart (54,84). Thus, TCDD can activate both detoxification signaling and hypertrophic signaling (85,86). Therefore, we further tested TCDD (10 nM) treatment in H9c2 cells for a short time of 4 h. Strikingly, this 4-hour treatment failed to generate hypertrophic response in the cell unlike 48-, or 72-hour treatment, and instead NQO1 level was stimulated (Supplementary Figure S5A-C). These results demonstrate that NQO1 expression is reduced only under TCDD-induced hypertrophic state in the cardiomyocyte. Concomitantly, TCDD treatment (100 nM for 24 or 48 h) that stimulated NQO1 expression in HEK 293 cells (Figure 1E) also did not induce hypertrophy in H9c2 cells, and instead there was increased NQO1 expression as in the case of HEK 293 cells (Supplementary Figure S5A-B, D) indicating the stimulation of detoxification pathway by TCDD under this condition. At present, how different concentrations of TCDD and duration of treatment induce either hypertrophic signal or detoxification signal is unclear. However, it is clear that NQO1 expression is reduced upon hypertrophy-induction in cardiomyocytes similar to isoproterenol treatment as opposed to its induction during detoxification pathway.

Hypertrophy was then induced *in vivo* in the Wistar rat heart model by intra-peritoneal isoproterenol injection for one week (63) and monitored after heart transitioned into hypertrophy. Induced-hypertrophy was assessed by analysis of molecular markers of hypertrophy (*ANP, BNP, SERCA2A, α-MHC* and *β-MHC*) (Figure 4F-G), and structural and physiological parameters of the heart such as heart size, increase in relative heart mass (ratio of heart mass to body mass), cross section of the heart, echocardiogram, and measurement of diastolic interventricular septal thickness (IVSd), diastolic left ventricular posterior wall thickness (LVPWd), left ventricular ejection fractions (LVEF), and left ventricular fractional shortening (LVFS) (Figure 4H-M). There was an enhancement in the heart size (Figure 4H) with a concomitant increase in relative heart mass (Figure 4I), and ventricular wall thickness (Figure 4J) of isoproterenol-injected animal heart compared to the control animals. Echocardiogram of hypertrophic and control animal showed altered physiological parameters such as increased IVSd and LVPWd, and a decrease in ejection fractions LVEF and LVFS measures (Figure 4K-M) in isoproterenol-injected animals confirming hypertrophic state of the animal heart (10,87).

**Figure 5.**
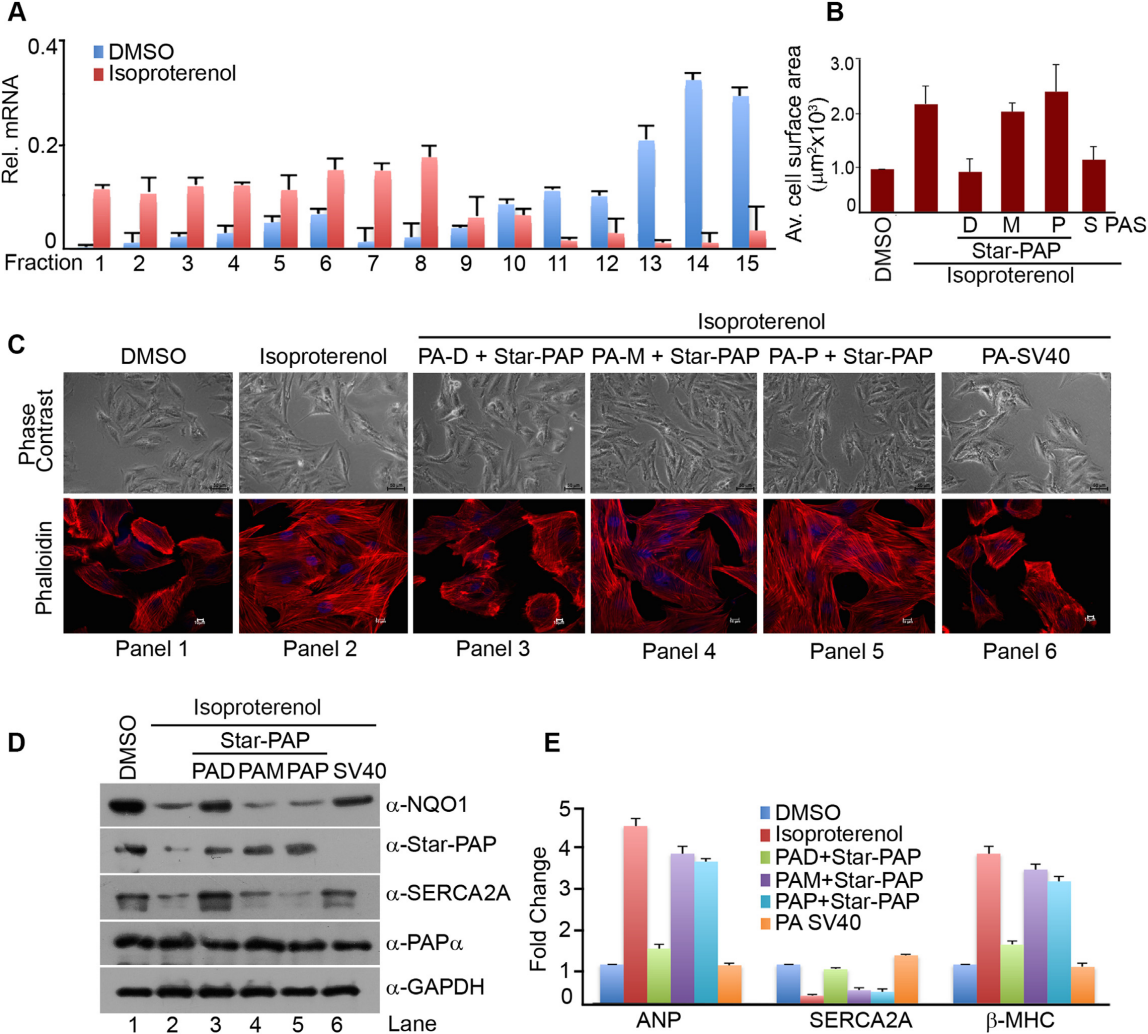
Ectopic expression of the distal-specific NQO1 isoform in the presence of Star-PAP reverses molecular events of hypertrophy in cardiomyocyte. (**A**) qRT-PCR analysis of the distribution of *NQO1* mRNA expressed as percent of total mRNA relative to *GAPDH* mRNA in each fraction across all fractions in the sucrose density gradient. Error bar represents SEM of *n* = 3 independent experiments (*P*-values <0.04 of *NQO1* in all the fractions). (**B, C**) Phase contrast (PC) and IF imaging of phalloidin stained H9c2 cells after treatment with isoproterenol in the presence of ectopic expression of different constructs of *NQO1* isoforms and Star-PAP as indicated, and quantification of cell surface area of the phalloidin stained H9c2 cells (*P* value <0.01 for all conditions). Error bar represents SEM. (**D**) Western blot analysis of Star-PAP, NQO1, PAPα, and SERCA2A after treatment with isoproterenol in the presence of ectopic expression of different constructs of FLAG-NQO1 and Star-PAP as indicated. Each blot is representative of 3 independent experiments. (**E**) qRT-PCR analysis of hypertrophic markers (*ANP, SERCA2A* and *β-MHC*) in H9c2 cell line under conditions as indicated. (*P*-values *ANP* <0.05, *SERCA2A* <0.04, *b-MHC* <0.01). Error bar represents SEM, *n* = 3 independent experiments.

As in the case of H9c2 cells, a reduction of both Star-PAP and NQO1 was observed in the left ventricular tissues from the hypertrophic Wistar rat heart in both Western and qRT-PCR analysis (Figure 4F-G) with concomitant reductions of SERCA2A, α-MHC or stimulation in ANP, BNP and β-MHC. There was also a progressive decrease in both NQO1 and Star-PAP levels with the duration of hypertrophic induction from early to late hypertrophy in the animal heart (Supplementary Figure S5E). The three *NQO1* APA isoforms were then assessed by 3′-RACE assay, and a selective reduction was observed of the distal-specific isoform during hypertrophy in both isoproterenol induced hypertrophy in H9c2 cells and animal heart tissues with no effect on the two shorter *NQO1* isoforms encoded from the middle- and proximal-sites (Figure 4N, Supplementary Figure S5F) as in the case of HEK 293 cells. Sequence comparison of human and rat 3′-UTR is shown in Supplementary Figure S6 with marked Star-PAP binding regions upstream of the distal PA-site on each sequence. To understand the link between NQO1 down regulation during CH and the loss of the distal PA-specific isoform, we analyzed the polysome profile of H9c2 cells in the presence and absence of isoproterenol treatment and measured the *NQO1* mRNA distribution across the polysome fractions of the sucrose density gradient as described in earlier sections (Figure 4O-Q). As in the case of HEK 293 cells, *NQO1* mRNA was largely distributed in the polysome portion of the gradient with >70% of the total NQO1 mRNA detected along with the polysomes (Figure 4P, R). Strikingly, there was reduction in the polysomal association of the *NQO1* mRNA on isoproterenol treatment similar to Star-PAP knockdown with only ∼15% of *NQO1* mRNA remaining in the polysome fractions (Figures 4R, 5A). This is consistent with the loss of the distal specific isoform during hypertrophy. These results indicate that reduced NQO1 protein expression is associated with a loss of the distal-specific isoform during cardiomyocyte hypertrophy. Previously, we reported a loss of Star-PAP along with an associated protein RBM10 during CH and established Star-PAP as a key cardiac PAP that controls anti-hypertrophy regulators in the heart (10). Together, our results demonstrate that the loss of NQO1 expression during CH involves *NQO1* APA where distal PA-site specific transcript is reduced as a result of inherent down regulation of Star-PAP during CH.

Strikingly, re-expression of the distal-specific *NQO1* isoform rescued the loss of NQO1 expression during isoproterenol-induced hypertrophy in H9c2 in the presence of exogenous Star-PAP expression (Figure 5D). Expression from the proximal- or middle-specific constructs failed to rescue the expression under similar conditions. Nevertheless, expression from the control *SV40*-specific *NQO1* constructs rescued the loss of NQO1 during isoproterenol-induced hypertrophy in H9c2 cells (Figure 5D). The same re-expressions of the *NQO1* distal-specific isoform together with Star-PAP also resulted in a significant reversal of cellular and molecular events of hypertrophy in H9c2 cells (Figure 5B-E). At the cellular level, increased cell size observed on isoproterenol treatment was reduced to normal size on the distal specific *NQO1* expression (Figure 5B-C). While *ANP*, or *β-MHC* up regulated on isoproterenol treatment were significantly attenuated, and SERCA2A expression was restored to normal levels in both Western and qRT-PCR analysis (Figure 5D-E). However, expression of proximal-, middle-, or distal-specific isoforms in the absence of Star-PAP did not rescue loss of NQO1 or isoproterenol-induced hypertrophy (data not shown). Together, our results indicate that hypertrophic signal induced down regulation of Star-PAP diminishes distal PA-site usage in *NQO1* APA, and this accounts for compromised NQO1 protein expression in CH. Our results establish a novel APA mechanism mediated via Star-PAP PA-site selection coupled differential PA-tail length addition, with implications in heart failure.

## DISCUSSION

**Figure 6.**
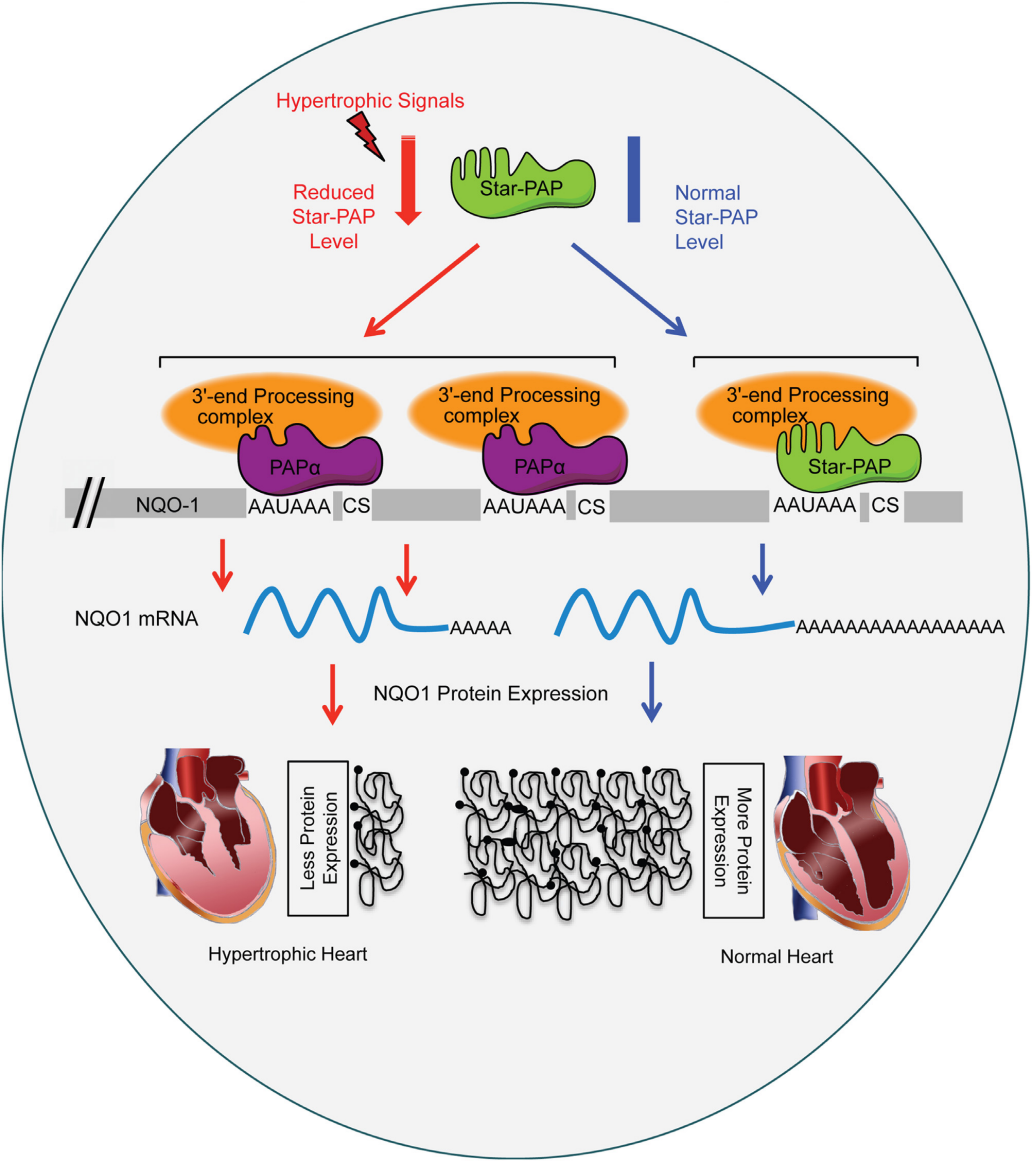
Mechanism of hypertrophic signal mediated alternative polyadenylation operated through Star-PAP at the *NQO1* UTR.

Alternative polyadenylation is a widespread mechanism, processing over 70% of human mRNAs (14,15,88). Yet, the mechanism of APA regulation and its function in gene expression are still emerging. Here, we show a new mechanism of APA operating at least at the *NQO1* 3′-UTR that is critical for protein expression during CH, an antecedent condition to incident HF. In case of *NQO1* mRNA, APA mediated 3′-UTR shortening causes reduced NQO1 protein expression during CH. A model summarizing these results and mechanism is shown in Figure 6. Earlier studies have reported widespread shortening of mRNA 3′-UTRs during CH that results from a hypertrophy-induced APA-shift towards proximal PA-site (22,24). This 3′-UTR shortening reduces overall miRNA interactions with the APA isoform, which in turn induces expression of pro-hypertrophic genes in CH (22,24). However, many mRNAs that exhibit shortened 3′-UTR during hypertrophy, e.g. kruppel like factor 4 (KLF4), myeloid ecotropic viral integration site 1 (Meis1), diacylglycerol kinase ϵ (DGKE), or uncoupling protein 3 (UCP3) (24) are anti-hypertrophy regulators that are down regulated during CH (89–92). The discrepancy in this mechansim of hypertrophy-induced APA and resultant protein expression during CH is unclear. It is possible that APA on the 3′-UTRs of these mRNAs (some, if not all) have mechanism similar to that in *NQO1* 3′-UTR shortening during CH.

We have reported Star-PAP as the key PAP that controls expression of anti-hypertrophic regulators in the heart and there is inherent depletion of Star-PAP during CH (10). Consequently, several anti-hypertrophic factors are downregulated during CH (93). *NQO1* is one such downregulated transcript under the regulatory control of the transcription factor Nrf2 (50,51). Downregulation of NQO1 expression during CH is associated with the loss of the longer *NQO1* transcript specific to the distal-most PA-site. This finding is in stark contrast to the prevailing view that the shorter mRNAs from APA generates more proteins compared to the longer transcript due to the loss of potential miRNA binding sites on the shortened 3′-UTR (21–23). We have defined a mechanism where differential PA-tail length at the 3′-end determines levels of proteins encoded by distinct *NQO1* APA isoforms during CH. The *NQO1* longest transcript regulated by Star-PAP has intrinsically longer A-tail compared to a short A-tail added on the two other shorter transcripts regulated by PAPα. But, it is not clear how differential (A) tail additions are controlled at the 3′-end of *NQO1* APA isoforms. Star-PAP or PAPα are unlikely to play roles in the PA-tail length determinations of the different *NQO1* isoforms. The two PAPs show robust and similar activity toward universal polyadenylation template. Moreover, PAPα target mRNAs such as *GAPDH* or *GCLC* have regular PA-tail length at the 3′-end similar to Star-PAP targets such as *HO-1* or *NQO1*. Therefore, the mechanism of PA-tail length control is likely more complicated than the current view involving additional components with possible impacts from cellular signals such as hypertrophic signal at the *NQO1* UTR or other similar mRNAs (75,94).

PA-tails are bound by PA-binding proteins (PABP) and it has been established that both the length of the PA-tail and its binding to PABP is critical for the translation of eukaryotic mRNAs (75,95). The earlier view that longer A-tails led to an increased translation efficiency (75,76), was supported by experiments using *in vitro* translation systems, or cellular reporter assays (61,80). However, with emerging studies, coupling of translation efficiency with PA-tail length has become a matter of debate (77–79,96,97). Two new studies have challenged the old view on the relationship between PA-tail length and translational efficiency using high throughput PA-tail sequencing in non-embryonic cells (77,78). Recently, Park *et al.*, revisited and established the earlier view of coupled PA-tail length and translation efficiency but within a limited range of A-length for cell cycle regulated mRNAs (79). Further, Lima *et al.*, demonstrated a direct link between the PA-tail length and the translation efficiency of mRNAs in *Ceanorhabditis elegans* (97). They observed the shorter A-tails (∼70 nt median tail length) were more associated with active/higher expressed genes compared to the longer A-tail (>95 nt median length). Thus, these studies indicated an optimal and a well-defined PA-tail length for efficient translation of mRNAs (96,97). In our study, the *NQO1* longest isoform had ∼70 nt median length that ranged from 50–90 nts. This would correspond to the optimal tail length required for efficient translation. The proximal or middle isoforms had less than or close to 20 nt, this would likely be sub-optimal for PABP binding and translation. This is consistent with the observations of well-defined PA-tail length and their correlation with translation state of mRNAs in the cell (79,97). One related example is that of CPEB-mediated cytoplasmic polyadenylation-induced translation activation in early development, cellular senescence, cell cycle progression, and synaptic stimulations (98–100). CPEB by virtue of its interaction with both cytoplasmic PAP (hGld2) and deadenylase PARN keeps the PA-tail on CPE-containing mRNAs relatively short (∼20–40 nucleotides) rendering the mRNA translationally inactive. Translation activation of these mRNAs occurs when PARN is expulsed from the 3′-UTR upon CPEB phosphorylation downstream of the stimulation signals leading to hGLD2-mediated PA-tail elongation (∼150 nucleotides) of the mRNA targets (76,99,101). Nevertheless, we have confirmed the effect of longer PA-tail length on *NQO1* mRNA isoform using an *in vitro* translation system. These data support the emerging concept of the existence of an optimal PA-tail length for efficient translation of mRNAs.

Another key question is the mechanism of APA and the role of PAPs in the PA-site selection. A number of factors including several core processing and RNA binding proteins such as CstF-64, U1 snRNP, PABPN1, hFIP1, and CF Im are reported to regulate APA site selection (31–36). Earlier, we showed that distinct PA-site usage at a genome-wide level by different PAPs: PAPα, PAPγ and Star-PAP (44). For *NQO1* APA, Star-PAP selects the distal site while the middle and proximal sites are regulated by the canonical PAPα consistent with our model of PAP-mediated PA-site selection to regulate APA. How do PAPs select target PA-site(s)? We reported a GC-rich sequence with an -AUA- motif for Star-PAP recognition, and a suboptimal downstream region with a U-depleted sequence that serves to exclude PAPα from Star-PAP targets (9,57). The *NQO1* distal site has all the elements required to qualify as a Star-PAP target PA-site, and altering this specific 3′-UTR sequence switches the regulatory PAP from Star-PAP to PAPα suggesting a sequence-specific selection of PA-sites by PAPs (1,57). Interestingly, Star-PAP selection of *NQO1* distal site is signal regulated. At least two agonists—oxidative stress and the toxin dioxin regulate the distal PA-site selection by Star-PAP. This indicated that diverse cellular signaling events influence the Star-PAP mediated-APA to control distinct cellular functions. For example, association of Star-PAP with kinases CKIα regulates cell invasion and oxidative stress, PKCδ association regulates apoptosis and DNA damage (5,9,11). Therefore, in addition to the sequence elements, APA is regulated through signaling molecules, and signal regulated PAP associated factors are likely to be involved in the selection of distinct PA-site(s) under different cellular conditions.

## Supplementary Material

gkz875_Supplemental_File
