# Supplementary material for: Star-PAP controlled alternative polyadenylation coupled poly(A) tail length regulates protein expression in hypertrophic heart
Source: Nucleic Acids Res. 2019 Oct 10;47(20):10771–87. doi: 10.1093/nar/gkz875 (PMC6847588; doi:10.1093/nar/gkz875)
Supplement: gkz875_Supplemental_File [file gkz875_supplemental_file.pdf]

Supplementary Data

Title

Star-PAP controlled Alternative Polyadenylation coupled PA-tail length regulates protein expression in Hypertrophic heart

Supplementary Figure Legends:

Supplementary Figure 1: UTR sequence of respective *NQO1* PA-site (proximal, distal and middle) and control *SV40* UTR. PA-site, cleavage site, Star-PAP binding motif, and downstream sequence along with various mutations introduced are indicated.

Supplementary Figure 2:(A-D) 3′-RACE assay of Star-PAP target mRNAs with multiple PA-sites (*PTBP2, ANXA7, FOG2, PAK1*). (E) 3′-RACE assay of *NQO1* UTR or control *GAPDH* UTR in the presence of CKIα knockdown after treatment of tBHQ, TCDD or DMSO as indicated. (F) Western blot analysis of NQO1 protein under conditions as in E. Each blot is representative of n=3 independent experiments. (G) 3′-RACE assay of *NQO1* after transfection with various NQO1 mini-gene reporter constructs (proximal, middle, distal and *SV40* driven) in the presence or absence of Star-PAP knockdown and treatment with tBHQ (100 μM for 4 hour) or TCDD (100 nM for 24 hours). (H-I) Western blot analysis of FLAG NQO1 after transfections of various reporter constructs as indicated as in G.

Supplementary Figure 3: (A) RNA sequence analysis upstream of each PA-sites on *NQO1* UTR. Reg RNA2 was used to scan for regulatory sequences and miRNA sites were probed using miRDB. Key sequence element considered that is likely to affect mRNA stability at the UTR (RBS-ribosome binding site, ARE-AU rich element, UTRM-UTR motif (that includes mushashi element, PIWI RNA and more), RES-RNA editing site, RS-riboswitches, LS-long stem, RHR-ncRNA hybridisation region) is indicated. (HSS-human splice site, ESE-exon splicing enhance, ESS-exon splicing silencer, ISE-intron splicing enhancer, ISS-intron splicing silencer). Potential miRNA sequences and PIWI motif detected are shown below. (B) Agarose gel analysis of residual RNA templates of short and long A tailed *NQO1* RNA after in vitro translation as in Figure 2M. (C) Western blot analysis of

FLAG NQO1 after treatment of reporter *NQO1* distal PA-site specific construct transfected HEK 293 cells with 100 μM cordycepin for various time points as indicated.

Supplementary Figure 4: (A) Analysis of GC and AT composition at *NQO1*, *BIK*, and *GCLC* UTRs upstream of the PA-signal as indicated. (B) Analysis of nucleotide composition (U, G, A and C) downstream of each *NQO1*, *BIK*, *GCLC* and *GAPDH* PA-sites. (C) qRIP analysis of PAP occupancy around the PA-sites within the target mRNA 3′-UTR. The primer sets used for qRIP are illustrated as arrows. The expression of the ORF of target genes was used as internal control (Internal). Error bars represent standard error of the mean of 3 independent experiments with triplicates for each experimental condition. (D-E) Quantification and the in vitro cleavage assay with nuclear extracts after control or siRNA Star-PAP knockdown. Relative intensities (in arbitrary units) for cleaved fragments were expressed as fraction cleavage relative to total intensity of both uncleaved and cleaved fragments. Error bar represents SEM, n=3 independent experiments. Upstream fragment (Cup) and downstream fragment (Cdn) after the cleavage of the template are indicated.

Supplementary Figure 5: (A) Phase contrast (PC) and IF imaging of phalloidin stained H9c2 cells after treatment with TCDD at various time points with two different concentrations (10 nM and 100 nM) as indicated along with control isoproterenol (100 μM for 48 hours)to test induction of hypertrophy. Quantification of cell surface area of the phalloidin stained H9c2 cells are shown B. Average cell surface area was measured for >50 cells per experiment for n>3 independent experiments (p value - 0.001 for DMSO, Isoproterenol, 4 hr TCDD (10 nM), and 0.005 for 88 hours and 72 hours respectively with TCDD 100 nM. Error bar represents SEM. (C-D) Western blot analysis of various hypertrophic markers, and NQO1 after TCDD treatment for different time points as indicated as in A. Each blot is representative of n = 3 independent experiments. (E) Western blot

analysis of Star-PAP, NQO1 and molecular marker SERCA2A from Wistar rat heart at progressive time points of hypertrophic induction (early, mid and late hypertrophy). (F) 3′-RACE assay of *NQO1* APA from control and hypertrophic heart tissue from Wistar rat as in Fig. 4F.Each gel is representative of n=3 independent experiments.

Supplementary Figure 6: Sequence comparison of the *NQO1* 3′-UTR regions of human and rat. Three NQO1 PA-signals (proximal, middle, and distal), along with the Star-PAP binding sites upstream of the distal PA-site in both rat and human are indicated.

List of antibodies:

Rabbit monoclonal anti-FLAG (Sigma), Rabbit monoclonal anti-β-Tubulin (Santacruz), Rabbit polyclonal anti-PIPKIα (Mohan *et. al.* 2015), Rabbit polyclonal anti-Star-PAP (Mohan *et. al.* 2015), Rabbit polyclonal anti-PAPOLA (Bethyl lab), Rabbit polyclonal anti-PAPOLG (Bethyl lab), Mouse monoclonal anti-NQO1 (Novus), Mouse monoclonal anti-SERCA2A (Santa Cruz), Goat polyclonal anti-ANP (Santacruz), Mouse monoclonal anti-GAPDH (Santa Cruz), Rabbit polyclonal anti-RNA Polymerase II (Bethyl lab) and Rabbit polyclonal anti-CKIα (Bethyl lab), and Mouse monoclonal anti-SKA (Santa Cruz) were used for Western blot, IP or RIP experiments.

List of Primers:

qRT-PCR (Human):

1. *NQO1* FP: 5′-GAACTTCAATCCCATCATTTCCAG-3′
   RP: 5′-CAGCTTCTTTTGTTCAGCCACAAT-3′
2. *FLAG-NQO1* FP: 5′-ATGGATTACAAGGATGACGACG-3′
   RP: 5′-CCTCCTTCATGGCATAGTTGAA-3′

3. *GCLC* FP: 5′-AAGTTCTTGAAACTCTGCAAGAGAAGG-3′

RP: 5′-GCCTCAACTGTATTGAACTCGGAC-3′

4. *GAPDH* FP: 5′-GAAGGTCGGAGTCAACGGATTT-3′

RP: 5′-GAATTTGCCATGGGTGGAAT-3′

qRT-PCR (Rat):

1. *ANP:* FP: 5′- TATACAGTGCGGTGTCCAAC-3′

RP: 5′-TCTCTGAGACGGGTTGACTT-3′

2. *BNP:* FP: 5′-AACAATCCACGATGCAGAAG-3′

RP: 5′-GTGCCATCTTGGAATTTCG-3′

3. *SERCA2A* FP: 5′-TTCGAAGTCTGCCTTCTGTG-3′

RP: 5′-CTCCAATGGGTGCATAGGTT-3′

4. *β-MHC* FP: 5′-ACAAGTTTGGCCACACCAA-3′

RP: 5′-TGTTCCACTGGATAATCAGCA-3′

5. *NQO1* FP: 5′-AAGGCTGGTTTGAGAGAGTG-3′

RP: 5′- ATTGGCCAGAGAATGACGTT-3′

6. *Star-PAP* FP: 5′-AGACCCACAGACTCCAAAGC-3′

RP: 5′-GTCAGAGCCCAAAGCAGAGT-3′

7. *GAPDH* FP: 5′-CCATGTTTGTGATGGGTGTG-3′

RP: 5′-ACAGTCTTCTGAGTGGCAGTGA-3′

9. *α-MHC* FP: 5′- TGCGGGTGAAGAAGAAGA-3′

RP: 5′- TCTCCTTCAGGTCGTCATTG-3′

3′- RACE (Human):

1. *NQO1* 5′-AACTTCCAGGCAGGATTCTTA-3′

2. *GAPDH* 5′-TTTGGCTACAGCAACAGGGT-3′

3. *ANXA7* 5′-TCCGGAATCCCTCTAAGTCT-3′

4. *PAK1* 5′-GGCTCTGTCAAGCTAACTGA-3′
5. *FOG2* 5′-GGCACAGTCTAAATCGAAAC-3′
6. *PTBP2* 5′-CCTTGCATTGTAATATTCAGTTT-3′

3′- RACE (Rat):

1. *NQO1* 5′-GTGTACAGCATTGGCCACA-3′
2. *GAPDH* 5′-GTGGACCTCATGGCCTACA-3′

Polysome Profiling (Human):

1. *NQO1* FP: 5′-GAACTTCAATCCCATCATTTCCAG-3′
   RP: 5′-CAGCTTCTTTTGTTCAGCCACAAT-3′
2. *FLAG-NQO1*FP: 5′-ATGGATTACAAGGATGACGACG-3′
   RP: 5′-CCTCCTTCATGGCATAGTTGAA-3′
3. *GAPDH* FP: 5′-GAAGGTCGGAGTCAACGGATTT-3′
   RP: 5′-GAATTTGCCATGGGTGGAAT-3′

Polysome Profiling (Rat):

1. *NQO1* FP: 5′-AAGGCTGGTTTGAGAGAGTG-3′
   RP: 5′- ATTGGCCAGAGAATGACGTT-3′
2. *GAPDH* FP: 5′-CCATGTTTGTGATGGGTGTG-3′
   RP: 5′-ACAGTCTTCTGAGTGGCAGTGA-3′

In vitro cleavage (Human):

1. *NQO1* Clv FP: 5′-TGCCTTCATCTTCACTGCAA-3′
   Clv RP: 5′-TTGTCAAGCCAGTCACCAAG-3
2. *GCLC* Clv FP: 5′-ATGCCTGGTTTTCGTTTGCA-3′
   Clv RP: 5′-AGCTGTGGAACTCACACACACTCA-3′
3. *GAPDH* Clv FP: 5′-CACACTGAATCTCCCCTCCT-3′

Clv RP: 5′-TTGACACAAGCCCAGCTTC-3′

cRACE and Sequencing:

1. *NQO1*-outer FP 5′-ACTCACTCGGTCATGCTAGT-3′

   RP 5′-GCTCGACCTTTTGGAGGAAA-3′
2. *NQO1*-inner FP 5′-AGGCCGACGTTGGAACACCC-3′

   RP 5′-TATAAAACAACGGAAGTAGA-3′
3. M13 Sequencing 5′ CAGGAAACAGCTATGAC-3′

In vitro translation:

1. *NQO1* FL FP:5′-GGCAAGCTTCGCCACCATGGATTACAAG-3′

   RP: 5′-CCCGGATCCACTAGTTTTTTTTTTTTTTTG

   GTACCTTAATTAATTAAGGTA-3′
2. *NQO1* TRNC RP: 5′-CCCGGATCCACTAGTTTTTTTTTTTTTTT

   CACCAGTGGTGATGGAAA-3′

siRNA Oligos:

1. Control scrambled non-targeting :AGGUAGUGUAAUCGCCUUG
2. Star-PAP :GUGUGUUUGUCAGUGGCUU
3. PIPKIα : GAAGUUGGAGCACUCUUGG
4. CKIα : On target Smart pool (Dharmacon)
5. PAPα : GGAGACUGACUGCGUACUU
6. PAPγ :On target Smart pool (Dharmacon)

Supplementary Figure 1

Poly A Distal UTR

-120 ----------------------------------Star-PAP binding region

CACUGGUGGUUUUGCUCUCGACAGUAUCCACA*AUAGCUGACGGCUGGGUGUUUCAGUUUGAAAAUA*

(USE) --- -60 Binding motif

*UUUUGUUGCCUUCAUCUUCACUGCAA*UUUUGUGUAAAUUUCUCAAAGAUCUGAAUUAAAUA*AAUAAA*

CS ----------------Sub optimal DSE--------------- PAS

AUUCAUUUCUACAGACCCAC*A*CUCAUUGGUAUCAGUGAAGCUAAUGAUAGUGGAAUCUAGUGGGAGG

Poly A Distal Mutation

-120 ----------------------------Star-PAP non-binding region

CACUGGUGGUUUUGCUCUCGACAGUAUCCACA*AUAGCUGACGGCUGGGUGUUUCAGUUUGAAAAUA*

(USE) --- -60 PAS

*UUUUGUUGCCUUCAUCUUCACUGCAA*UUUUGUGUAAAUUUCUCAAAGAUCUGAAUUAAAUA*AAUAAA*

CS --------DSE with CstF binding region-------

AUUCAUUUCUACAGACCCAC*A*CUCAUUGGUAUCAGUGAAUUUUUUGAUAGUGGAAUCUAGUGGGAGG

Poly A Proximal UTR

-120 -----------------------Equivalent USE (Star-PAP non-binding)------

UAGAAAAUGAGAUUCCTTAGCCUGG*AUUUCCUUCUAACAUGUUAUCAAAUCUGGGUAUCUUUCCAGG*

-------------------------------- -60 PAS

*CUUCCCUGACUUGCUUUA*GUUUUUAAGAUUUGUGUUUUUCUUUUUCCACAAGG*AAUAAA*UGAGAGGG

CS ------------------------DSE with CstF binding region-----------------------

AAUCGACUGU*A*UUCGUGCAUUUUUGGAUCAUUUUUAACUGAUUCUUAUGAUUACUAUCAUGGCAUAU

Poly A Proximal Mutation

-120 -----------------------Equivalent USE (Star-PAP binding)-------------

UAGAAAAUGAGAUUCCTTAGCCUGG*AUUUCCUUCUAACAUGUUAUCAAAUAUUUUUAUCUUUCCAGG*

-------------------------------- -60 Binding motif PAS

*CUUCCCUGACUUGCUUUA*GUUUUUAAGAUUUGUGUUUUUCUUUUUCCACAAGG*AAUAAA*UGAGAGGG

CS ---------------------------------Sub optimal DSE--------------------------------

AAUCGACUGU*A*UUCGUGCAUUUUUGGAUCAUUUUUAACUGAUUCUUAUGAUUACUAUCAUGGCAUAU

Poly A Middle UTR

-120 ---------------------------------------Equivalent USE

AUUACAAAGCAGUUACUAAUAUGCCUAGCACAAGUACCACUCUUGGUCAGCUUUUGUUGUUGCACAA

(Star-PAP non-binding)------------------------ -60 PAS

*GUACCACUCUUGGUCAGCUUUUGUUGUUUAUAUACAGUA*CACAGAUACCUUGAAAGGAAGAGCUAAU

CS ---------------DSE with CstF binding region--------------

AAAUCUCUUCUUUGCUGCAGUC*A*UCUACUUUUUUUUUAAUUAAAAAAAAUUUUUUUUUGAAGCAGTCT

Poly A SV40 UTR

PAS

AAUGAAUGCAAUUGUUGUUGUUAACUUGUUUAUUGCAGCUUAUAAUGGUUACAAAUAAAGCAAAUAG

---DSE with CstF binding region---

CAUCACAAAUUUCACAAAUAAAGCAUUUUUU

Supplementary Figure 2

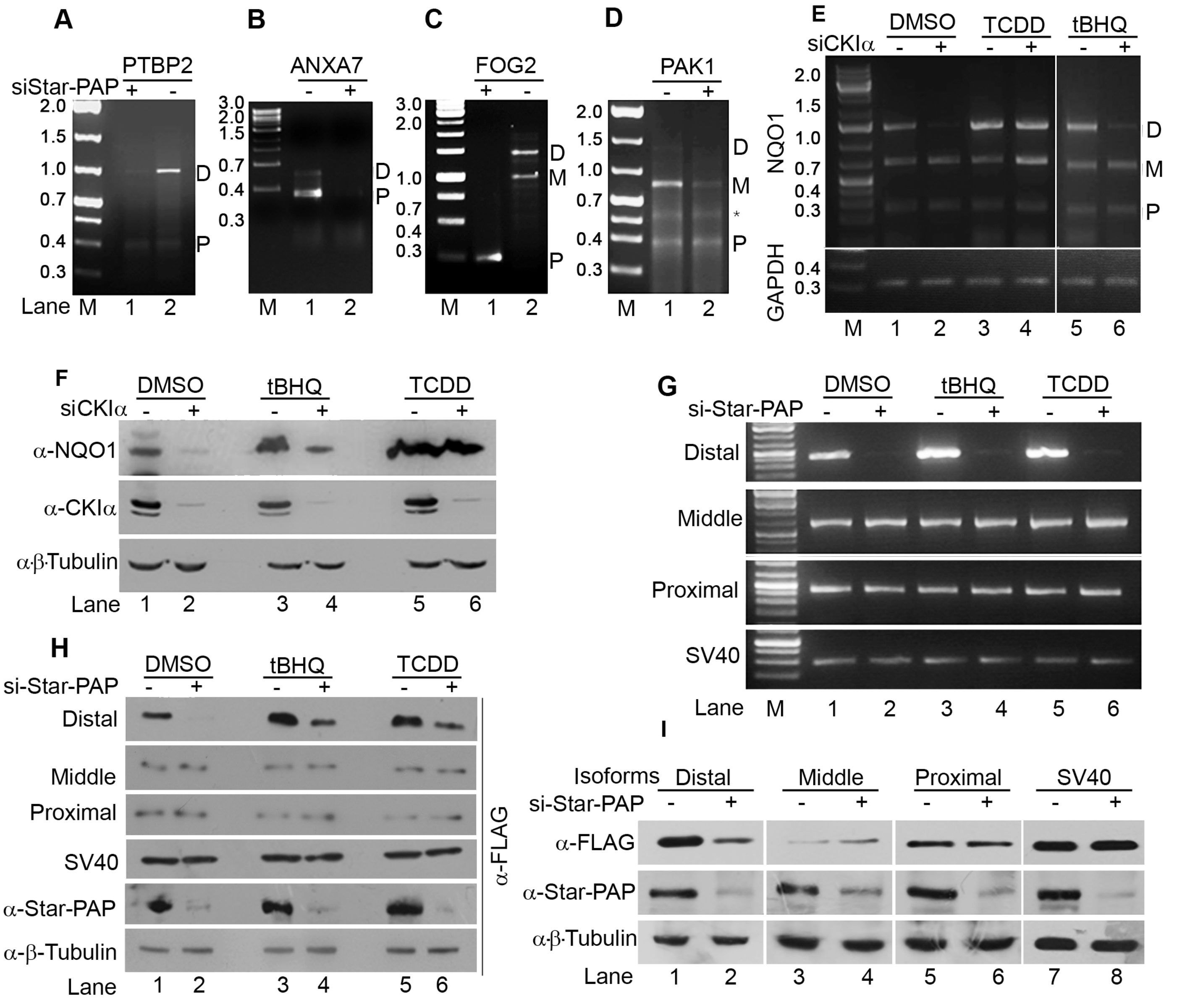

Supplementary Figure 3

A

RNA element analysis   miRNA scan: miRDB

RNA Motif search : Reg RNA 2 (doi:10.1093/nar/gkl333)

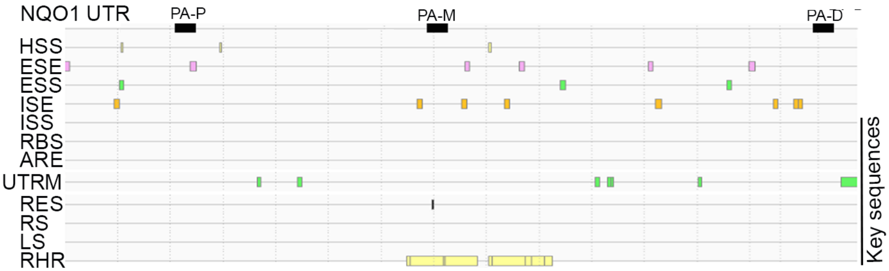

	Proximal	Middle	Distal
miRNA binding sites	1	6	4
AU rich elements	0	0	0
piwi interacting RNA	0	5	0

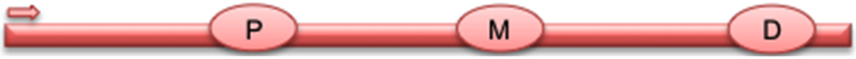

hsa-miR-6833-5p (98)

hsa-miR-5197-3p (97)
hsa-miR-18b-3p (97)
hsa-miR-1237-3p (94)
hsa-miR-3613-3p (84)
hsa-miR-2114-5p (82)
hsa-miR-3908 (81)

hsa-miR-6833-5p (98)
hsa-miR-1237-3p (94)
hsa-miR-4659b-5p (81)
hsa-miR-605-3p (82)

B

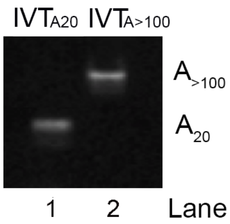

C

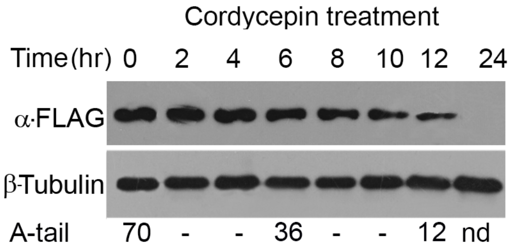

Supplementary Figure 4

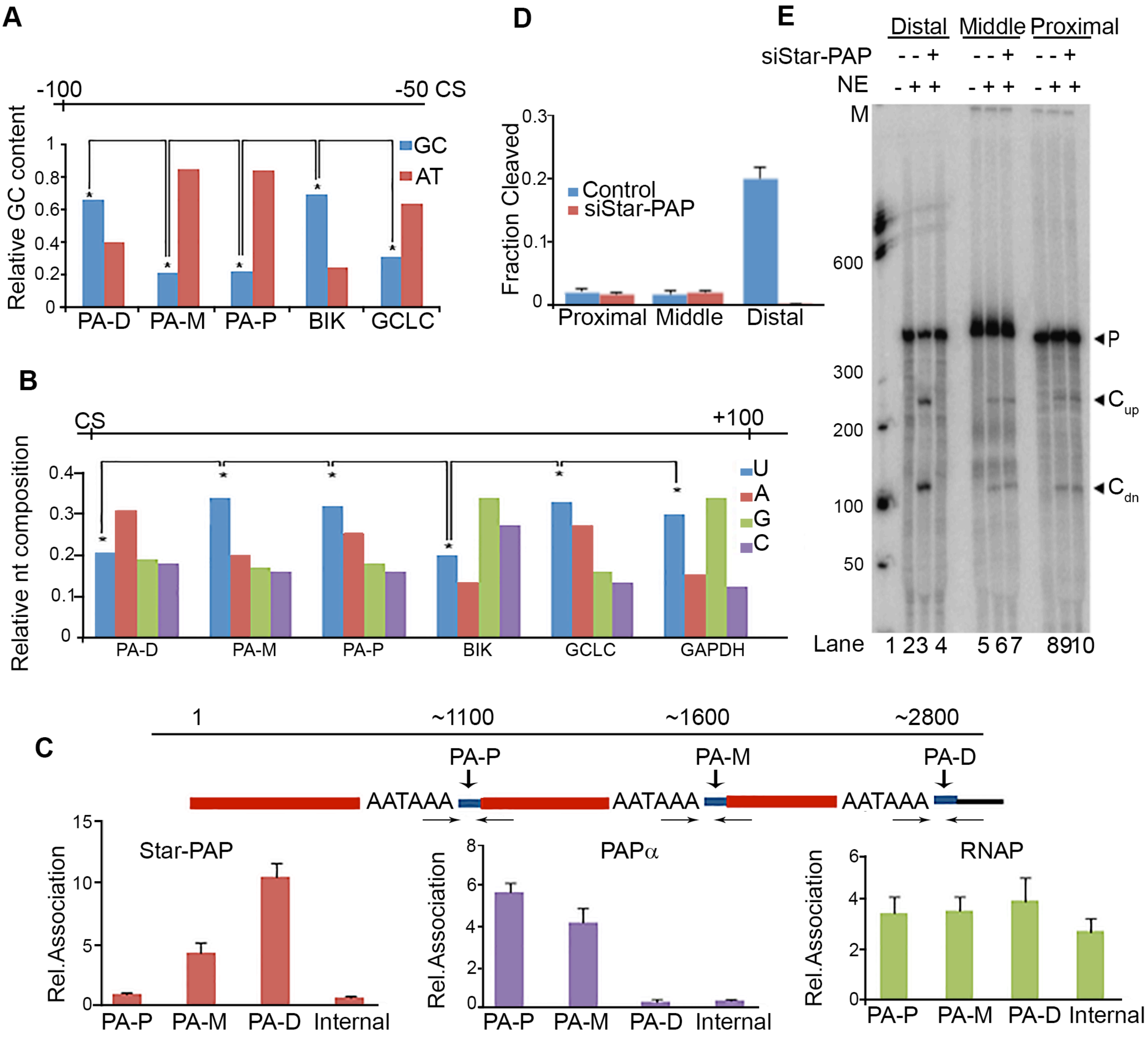

Supplementary Figure 5

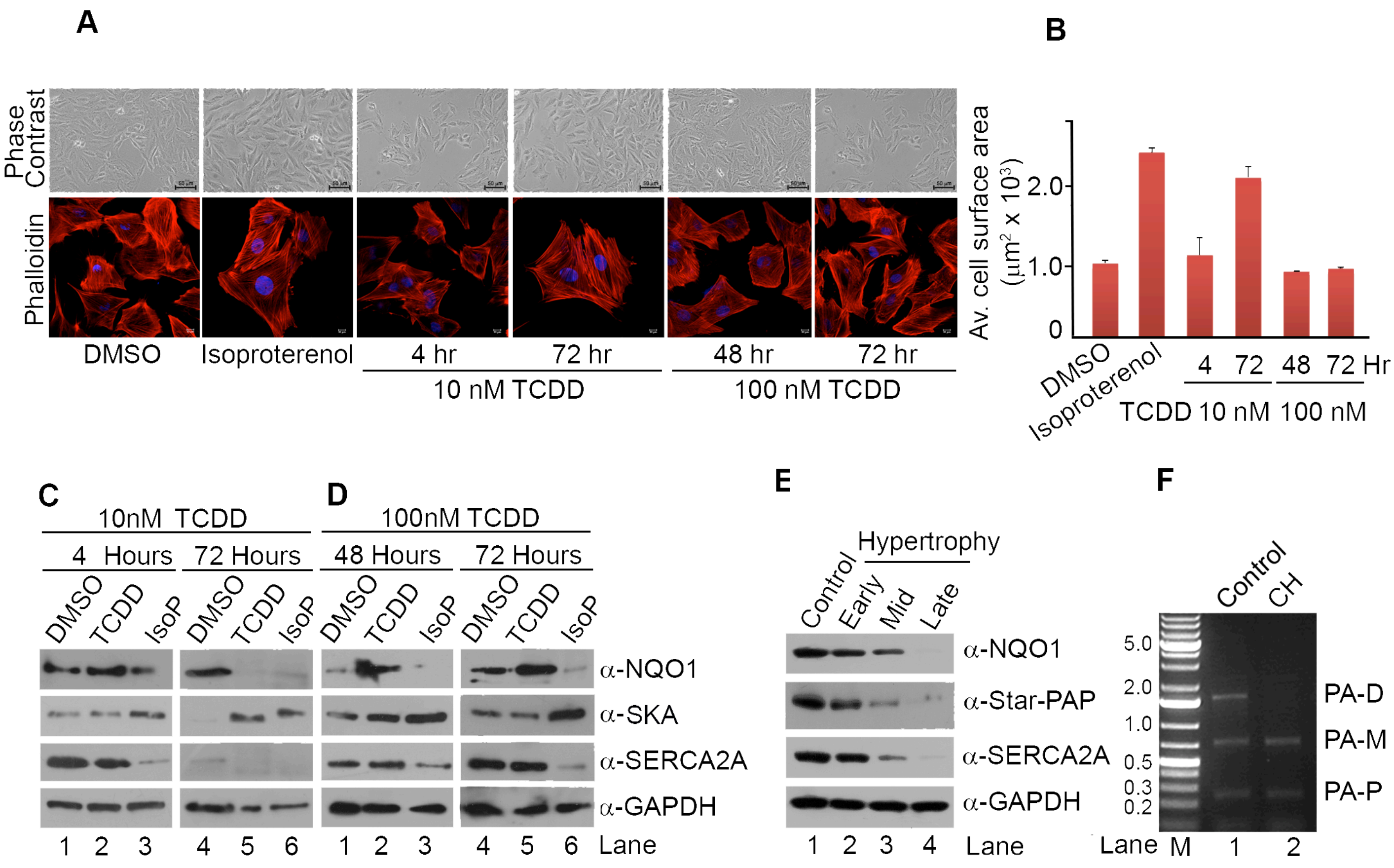

Supplementary Figure 6

```
1    GGAAGAAACGCCTGGAGAATATTTGGGATGAGACACCACTGTATTTTGCTCCAAGCAGCC  Hsapiens
1    GGAAGAAGCGTCTGGAGACTGTCTGGGAGGAGTCACCACTCTACTTTGCTCCAAGCAGCT  Rnorvegicus
     ******* ** ******* * * ***** *** ******* ** ***************

61   TCTTTGACCTAAACTTCCAGGCAGGATTCTTAATGAAAAAAGAGGTACAGGATGAGGAGA  Hsapiens
61   TGTTTGACCTAAACTTCCAGGCAGGATTCTTACTGAAAAAAGAGGTTCAAGAGGAGCAGA  Rnorvegicus
     * ***************************** ************* ** ** *** ***

121  AAAACAAGAAATTTGGCCTTTCTGTGGGCCATCACTTGGGCAAGTCCATCCCAACTGACA  Hsapiens
121  AAAAGAACAAGTTTGGCCTTTCTGTGGGCCATCATTTGGGCAAGTCCATTCCAGCCGACA  Rnorvegicus
     **** ** ** ********************* ************** *** * ****
                         Stop codon
181  ACCAGATCAAAGCTAGAAAATGAGATTCCTTAGCCTGGATTTCCTTCTAACATGTTATCA  Hsapiens
181  ACCAGATCAAAGCTAGAAAATAAGGTTTTCCATACCATGTAGTTAGACCCAGG-------  Rnorvegicus
     ******************** ** **    *  *    *
                         Stop codon
241  AATCTGGGTATCTTTCCAGGCTTCCCTGACTTGCTTTAGTTTTTAAGATTTGTGTTTTTC  Hsapiens
231  -----TTTCTTTTTCCCCAGTTTGCCTGGCTTGCTTTCAGTTTTCGCCTTTGT-------  Rnorvegicus
          * ** **  * ** **** ********   ****    *****
                Proximal
301  TTTTTCCACAAGGAATAAATGAGAGGGAATCGACTGTATTCGTGCATTTTTGGATCATTT  Hsapiens
282  -------TCCACAAGGATAGGAAAAGGAGGAGGCTCGCCTCATGCGTTTTTGGATAGTTC  Rnorvegicus
           * *  *  * * ** * ***   * **    ** *** *********  **

361  TTAACTGATTCTTATGATTACTATCATGGCATATAAC-CAAAATCCGACTGGGCTCAAGA  Hsapiens
335  TGCCACGGCTGTGACAGCAAAATGAACGAGGTCAGATTAGGGGCCTCAGGTGGCCTGGGA  Rnorvegicus
     *    *  * * *     *    * *   *    *          *  *   ***
                        Proximal                                **
420  GGCCACTTAGGGAAAGATGTAGAAAGATGCTAGAAAAATGTTCTTTAAAGGCATCTACAC  Hsapiens
395  TATGAATCAGGGAGAGGTGTAGCCGCGAGCGGGGAAAATACTCTTCTAGGTCTTTTGTAC  Rnorvegicus
         * * ***** ** *****      **  * *****  ****  * * * * *  **

480  AATTTAATTCCTCTTTTTAGGGCTAAAGTTTTAGGGTACAGTTTGGCTAGGTATCATTCA  Hsapiens
455  ACTATAAGCTTTTTTCTTCGGGCTA-------------GCCCTGGCTAAATGGCATCCA  Rnorvegicus
     * * ***    * ** ** ******                   ******  *  *** **

540  ACT------CTCCAATGTTCTATTAATCACCTCTCTGTAGTTTATGGCAGAAGGGAATTG  Hsapiens
501  ATCCTCCACCCACTTGTTGCTATTAGTTACCTCTCTGTGGTTTAGGGCAGGAGGGAATTG  Rnorvegicus
     *        *  *    * ****** * ********** ***** ***** *********

594  CTCAGAGAAGGAAAAGACTGAATCTACCTGCCCTAAGGGACTTAACTTGTTTGGTAGTTA  Hsapiens
561  CTCAAACAATGGCTGA--------------------GGGACTAACTTGTTTA----GCA  Rnorvegicus
     **** * ** *                    **   **********          *

654  GCCATCTAATGCTTGTTTATGATATTTCTTGCTTTCAATTACAAAGCAGTTACTAATATG  Hsapiens
596  GTTAGCTAAAGCCTGTTTATGATCCATCCTGGTTTCAATTACTGTGCAGTGACTGACAAG  Rnorvegicus
     *  * **** ** **********   ** ** **********   ***** *** * * *

714  CCTAGCACAAGTACCACTCTTGGTCAGCTTTTGTTGTTTATATACAGTACACAGATACCT  Hsapiens
656  CCTCGGGGGATTGCTCTCCAGCTCTTCTCTGCCTT--GTACATAGCACACCCAGGTCCTG  Rnorvegicus
     *** *    * * *   *         *   **   ** ***    ** *** * *
                  Middle
774  TGAAAGGAAGAGCTAATAAATCTCTTCTTTGCTGCAGTCATCTACTTTTTTTTTAATTAA  Hsapiens
714  GGAAATGA---ATACAAAAACAGGTCTCCGCCTCATTCTTGCTATTCTTTCTTTGTGTG  Rnorvegicus
      **** **     **  *** *   ***  **  ** ** *    ** *** **  *

834  AAAAAATTTTTTTTTGAAGCAGTCTTGCTCTGTTACCCAGGCTGGAG-----TGCAGTGG  Hsapiens
770  TGTGT--------GTGTGGAAATAAATGGATATTTCACACGTCAGATGGCGGTGCGTGCG  Rnorvegicus
               **  * * *       * ** * ** *   **      ***    *
                         Middle
889  TGTGATCTCGGCTCA---CTGCAACCTCTGCCTCCCAGGTTCCAGCAATTCTCCTGC-CT  Hsapiens
822  TTTGAGCCCTTCAATACTTAACAACAGCCCCCTCCTGAGCTGGCGCGATGACTCAGCAGC  Rnorvegicus
     * *** * *  *         ****  *  *****   * *   ** **     * **

945  CAGCCTCCCTAGTAGCTGGGATGACAGGCGCCTGCCATCATGCCTGACTAATTTTTGTAT  Hsapiens
882  TAAGAGCCCTTGTGGCT---CTAGCAGGAAACCCACCTAAAGGCTAACTGCTGTTCCTAA  Rnorvegicus
      *    **** ** ***     *  ****    *    * * * * ** ***  * **  **

1005 TTTTAGTAGAGAC---------GGCGTTTCACCATGTTGGCCAGGCTGGTCTCAAACTCC  Hsapiens
939  CTCCAGTTCTAAAGAACCCAGCGCCCTCTCCTGGTTTCTTCAGGCACTGCATGCAGGCAG  Rnorvegicus
      *  ***    *          * * * **    * *   *  *    *  *  *

     TGACCTCAGGTGATCCGCC---------------------TACCTCAGCCTCCCAAAGT  1093
     TGCACAGACATAACACGCGAGCCGAAAAATGGGTAGACACATACAAATAAATCCTGAACG  1058
     **  *  *  * *  ***                            ***        ***  **

     GCT---GGGATTACAGGCGTGATCCACCACACCTGGCCCTTGCAATCTTCTACTTTAAGG  1150
     CCAAAATGAAAACCAGTAGCCTTCTACCAAAC-----GTCTGGAAGGTTTTCTGTAGTA  1113
      *     * *  ***  *   ** **** **          ** **  ** * ** **

     TTTGCAGAGATAAACCAATAAATCCACACCGTACA-------TCTGCAATATGA------  1197
     TTGTGAAATCAATGACATTCCTTTCTTATTTATTTTATTTTTTTTGGTTCTTTTTTTCG  1173
     **   * *   *   ** *   * *  *   **             * *    * *

     --ATTCAAGAAAGGAAATAGTACCTTCAA-TACTTAAAAA--TAGTCTTCCACAAAAAAT  1252
     GAGCTGGGGACCGAACCCAGGGCCTTGCACTTCCTAGGCAAGCGCTCTACCACTGAGCTA  1233
         *   **  * *   **  ****  * * * **   *     *** ****  *

     ACTTTATTTCTGATCTATACAAATTTTCAGAAGGTTATTTTCTTTATCATTGCTAAACTG  1312
     AATCCCCAACCCCTCCTTTCTTATTTTAAAGAT-CTGTTTTGTTTTTAATTCTGTGGGTG  1292
     * *      *   **  * *  ***** *  *   * **** *** * ***       **

     ATGACTTACTATGGGATGGGGTCCAGTCCCATGACCTTGGGGTACAATTGTAAACCTAGA  1372
     GGG-----------AAGGGTGTGCGCGCGC----GCGTG-CGCGCGCACGCGTGCCTGAG  1336
       *          * ** ** *   * *     * **  *  *    *   ***

     GTTTTATCAACTTTGGTG-AACAGTTTTGGCATAATAGTCAATTTCTACTTC--------  1423
     GGTTTATGAATATGTGTGTACAAGTGCTCACAGACTCCTGAAGAAGGCCTTCCAGCCCCC  1396
     * ***** **  *  *** *  ***  *  ** * *  * **     ****

     --------------------------------TGGAAGTCATCTCATTCCACTGTTGGT  1450
     AGAGCTGGAGTTACAAGTGGCTGCTCTTAGCTATGAAACCATCTCCCCAGCCCTGATGAT  1456
                                        ** **       *    * *** ** *

     ATTATATAATTCAAGGAGAATATGATAAAACACTGCCCTCTTGTGGTGCATTGAAAGAAG  1510
     TACATTCAGTTTAAGGAAATATGCAGATATCATGTAGGTCTTTTTTTTTTTTTAATATTT  1516
        **  * ** ***** *     * * * **       **** *  *   ** **

     AGATGAGAAATGATGAAAAGGTTGCCT-GAAAAATGGGAGAC--------AGCCTC---T  1558
     ATTTTATGTA-GACAAGCATACTGTCTTCAAACACATCAAATCCCATTACAGATGGTTGT  1575
     *  * *   * **  *  *   ** **  *** *    * *          **       *

     TACTTGCCAAGAAAATGAAGGGATTGGACCGAGCTGGAAAACCTCCTTTACCAGATGCTG  1618
     GAGCCACCATGTGGTTGCTGGGATTTGAAC---------------------TCAGGACCTC  1615
      *    *** *    **  ****** ** *                      ***   **

     ACTGGCACTGGTGGTTTTTGCTCTCGACA-----GTATCCACAATAGCTGACGGCTGGGT  1673
     TCTGGAAGAGC-AGTCAGTGCTCTTAACCACTGAGCCATCTCTCTAGCCCTCATATAGGT  1674
      **** *  *   **   ******  **      *     * *  ****   *   * ***
                   Star-PAP binding motif
     GTTTCAGTTT---GAAAATATTTTGTTGCCTTCATCTTCACTGCAATTTTGTGTAAATTT  1730
     CTTATTATCTACAGTAAATAATTTTAAGACTGT-------------GTGTGTGTTGGTGT  1721
      **    * *   * ***** ***    * **                  * *****   * *
                       Distal
     CTCAAAGAT---CTGAATTAAATAAATAAAATTCATTTCTACAGACCCACACTCATTGGT  1787
     CTAATGAATAGTCTTACGAATGTGAAGCTAGCATCCTACTGTTGAACTACATTCTGCGTT  1781
     ** *   **    ** *   *  * **   *      * **   ** * *** **   * *
     Star-PAP binding motif
     ATCAGTGAAG---CTAATGATAGTGGAATCTAGTGGGAGGCAGATCAGAGGATTCAATCA  1844
     CCCAATTTTGGCATAAAATTAAGTGTTATTTAGGTGTGT------------------GTGC  1824
       ** *   *     **    ****  ** ***  *                    *
                   Distal
     CAAACTGCAGCTGGAAGCCCCTTGGTGACTGGCTTGACAACTCACCCAGCAGGGCTGCTT  1904
     ACGCGTGAGTGTGGCATGCCTGTAGAGACTAGAAGGCAGAGTCACATCTCCTGGCTGGAG  1884
          **    *** *  **   * * **** *   *   * ****    *  *****

     -----------------CCTACTAAATGGTA-ATAGAACAACCTATTCAGTCCTCTCAGG  1946
     TTACAGGCATTTGTAAGCCCTGTGAGTGCTCAGAATTGAACTCCACTTGGTCCTCTGCTA  1944
                         **   * * ** *    *    *  * * *  *******

     GAATTCAAAGGAAAGAGAGTGGTATAGAAATC--                           1978
     GAACAGCAAGGGCTTTTAAATGTTGGGCCACCTC                           1978
     ***    ****      *   **    *  * *
```
